# Study of the Therapeutic Effect of Cytokine-Preconditioned Mesenchymal Stem Cells and Their Exosomes in a Mouse Model of Psoriasis

**DOI:** 10.3390/biology14081033

**Published:** 2025-08-11

**Authors:** Aidar Dairov, Assel Issabekova, Madina Sarsenova, Aliya Sekenova, Miras Shakhatbayev, Symbat Alimbek, Gulshakhar Kudaibergen, Assiya Nurkina, Ilyas Akhmetollayev, Kyung-Sun Kang, Vyacheslav Ogay

**Affiliations:** 1 Stem Cell Laboratory, National Center for Biotechnology, Astana 010000, Kazakhstankudaibergen@biocenter.kz (G.K.); nurkina@biocenter.kz (A.N.); ogay@biocenter.kz (V.O.); 2 Department of General Biology and Genomics, L.N. Gumilyov Eurasian National University, Astana 010008, Kazakhstan; 3 Laboratory for Development of Molecular Diagnostic Approaches, National Center for Biotechnology, Astana 010000, Kazakhstan; 4 Adult Stem Cell Research Center, College of Veterinary Medicine, Seoul National University, Seoul 08826, Republic of Korea

**Keywords:** mesenchymal stem cell, human umbilical cord blood mesenchymal stem cell, exosome, cytokine, psoriasis, skin inflammation

## Abstract

Mesenchymal stem cells (MSCs) are a type of multipotent, non-hematopoietic cells of mesodermal origin. Due to their strong immunomodulatory, immunosuppressive, and regenerative potential, MSCs are used in cell therapy for inflammatory, immune-mediated, and degenerative diseases. Exosomes derived from MSCs have several advantages over MSC therapy, including non-immunogenicity, lack of infusion toxicity, ease of isolation, manipulation, and storage, cargo specificity, and the absence of tumor-forming potential and ethical concerns. We hypothesized that preconditioning human umbilical cord blood mesenchymal stem cells (hUCB-MSCs) with the proinflammatory cytokines interleukin 17 (IL-17), IL-22, and tumor necrosis factor alpha (TNF-α), the increased levels of which are typical in psoriasis patients, can significantly increase the therapeutic efficacy of both hUCB-MSCs and their exosomes (hUCB-MSC-Exo). Our aim was to compare the therapeutic effects of hUCB-MSCs preconditioned with various combinations of proinflammatory cytokines and their hUCB-MSC-Exo, in an in vivo imiquimod-induced psoriasis-like skin inflammation model in mice. Our results showed a significant attenuation of psoriasis symptoms (erythema, scaling, and skin thickness) in mice treated with intact hUCB-MSCs, hUCB-MSCs preconditioned with IL-22 and TNF-α, and hUCB-MSC-Exo preconditioned with IL-17, IL-22 and TNF-α (MSC-Exo 3C). However, the most pronounced therapeutic effect was observed with MSC-Exo 3C treatment. In summary, we demonstrated that MSC-Exo 3C transplantation has therapeutic potential for treating psoriasis-like skin lesions.

## Simple Summary

Mesenchymal stem cells (MSCs) are a type of multipotent, non-hematopoietic cells of mesodermal origin. Exosomes derived from MSCs have several advantages over MSC therapy, including non-immunogenicity, lack of infusion toxicity, ease of isolation, manipulation, and storage, cargo specificity, and the absence of tumor-forming potential and ethical concerns. Our aim was to compare the therapeutic effects of human umbilical cord blood MSCs (hUCB-MSCs) preconditioned with various combinations of proinflammatory cytokines elevated in psoriasis, as well as their exosomes (hUCB-MSC-Exo), in an in vivo imiquimod-induced psoriasis-like skin inflammation model in mice. We found a significant attenuation of psoriasis symptoms (erythema, scaling, and skin thickness) in mice treated with intact hUCB-MSCs, hUCB-MSCs preconditioned with interleukin 22 (IL-22) and tumor necrosis factor alpha (TNF-α), and hUCB-MSC-Exo preconditioned with IL-17, IL-22, and TNF-a (MSC-Exo 3C). However, the most pronounced therapeutic effect was observed with MSC-Exo 3C treatment. The data presented here suggest that subcutaneous administration of MSC-Exo 3C has therapeutic potential for treating skin inflammation and, thus, could have potential applications in psoriasis treatment.

## 1. Introduction

Psoriasis is a common chronic inflammatory multisystem disease that primarily affects the skin and joints and is associated with genetic predisposition and inflammatory dysregulation [1]. Approximately 2–3% of the world’s population suffers from this condition [2]. Race and geographic location are key factors influencing the prevalence of psoriasis. Psoriasis is most common in the European population in Western countries: Norway (4.6%), France (4.42%), Portugal (4.4%), and the United States (3.0%) [3]. This disease manifests as erythematous, itchy, scaly patches, and is characterized by a high incidence rate, long duration, and a tendency to relapse [4]. Psoriasis involves a complex interplay between immune cells, biological signaling molecules, and skin cells [5]. It is notably marked by activation and expansion of Th1, Th17, and Th22 T helper cell subsets [6]. These cells increase the production of cytokines in the skin, such as interferon gamma (IFN-γ), tumor necrosis factor alpha (TNF-a), interleukin (IL)-17, IL-22 and IL-23 [4,6]. Immunological and genetic studies have highlighted the critical role of the IL-23/IL-17 axis in driving the psoriatic inflammatory cascade [4,7]. Histologically, psoriasis is characterized by a thickening of the epidermis (acanthosis) with downward elongation of rete ridges, a granular layer that is either absent or thinned, elongated and dilated capillaries, suprapapillary thinning, and a dense infiltration of T cells in both the dermis and epidermis. Neutrophil aggregates within the parakeratotic stratum corneum may also be observed [4].

Modern treatment of psoriasis is aimed at reducing the symptoms of the disease and improving the quality of life of patients, including local therapy (vitamin D analogs, corticosteroids), phototherapy (narrowband ultraviolet-B radiation, psoralen, ultraviolet-A radiation), systemic immunomodulators (methotrexate, cyclosporine, acitretin), targeted biological agents (TNF-α, IL-17, IL-23 inhibitors), and oral small-molecule inhibitors (dimethyl fumarate, apremilast) [8,9]. However, side effects, treatment resistance, long-term safety concerns, and the high cost of some treatments limit their widespread use and effectiveness [8]. Consequently, there is a need to find alternative treatment strategies for psoriasis [10]. In recent years, novel therapeutic approaches and promising drug candidates have been actively explored in both preclinical and clinical studies [8]. Among these, mesenchymal stem cells-derived exosomes (MSC exosomes) have garnered considerable attention due to their potent immunomodulatory properties [10].

Mesenchymal stem cells (MSCs) are a type of multipotent, non-hematopoietic cells of mesodermal origin capable of proliferating and replacing damaged or dead cells within the organism [11]. MSCs have typical fibroblast-like morphology in culture. They are capable of differentiating into cell types of mesodermal (adipocytes, chondrocytes, osteocytes), endodermal (alveolar endothelial cells), and neuroectodermal (neuroglial cells) origin [12,13]. The robust immunomodulatory, immunosuppressive, and regenerative potential of MSCs has positioned them as a promising candidate for cell-based therapies in a variety of inflammatory, autoimmune, and degenerative diseases. [14]. Numerous studies have demonstrated the effectiveness of MSC therapy in the treatment of a wide range of pathological conditions, including cancer [15], atherosclerosis [16], rheumatoid arthritis [17,18], bone defects [19], skin wounds [20,21], and others. To date, the therapeutic properties of MSCs are being investigated in over 950 clinical trials worldwide [22].

The therapeutic effect of MSCs and their interaction with the immune system are mediated through both direct cellular contact and paracrine signaling [12]. Repair and regeneration of damaged tissues are facilitated by the migration and homing of MSCs to sites of injury, as well as their immunotropic properties. The mechanism of cell replacement therapy through direct differentiation of MSCs is also discussed [23]. MSCs secrete bioactive messengers (regulatory factors, chemokines, cytokines, growth factors, and nucleic acids) and package them into extracellular vesicles, including exosomes (MSC-Exo), a key mechanism in tissue repair [23,24]. MSCs produce trophic factors such as stromal derived factor-1 (SDF-1), hepatocyte growth factor (HGF), insulin-like growth factor (IGF-1), epithelial growth factor (EGF), nerve growth factor (NGF), transforming growth factor-alpha (TGF-a), and tissue angiogenesis vascular endothelial growth factor (VEGF) [25,26]. These bioactive factors have a variety of activities, including modulating the local immune response, enhancing angiogenesis, inhibiting cell apoptosis, and stimulating the survival, proliferation, and differentiation of resident tissue-specific cells [24]. However, there are factors limiting the widespread use of MSCs in clinical practice. These include the risk of tumor formation and transmission of viruses and prions after stem cell transplantation; loss of MSC multipotency during cultivation; low survival and engraftment rates of MSCs after transplantation; low therapeutic effect; and high production costs, as well as production requirements (GLP/GMP) [11,27,28,29,30,31].

Given these challenges, exosomes derived from MSCs (MSC-Exo), small, lipid bilayer-bound extracellular vesicles secreted by MSCs, have emerged as a promising alternative to direct MSC therapy. These nanosized vesicles (30–200 nm in diameter) can transport a variety of macromolecules, including nucleic acids (DNA, RNA, miRNA), membrane glycoproteins, lipids, and other cell-specific proteins, to neighboring or distant cells via endocytosis [32,33]. The unique features of exosomes include their large quantity in various body fluids, since healthy cells produce 10^3^–10^4^ exosomes per cell, and also the fact that they remain stable at −80 °C. They also have a long half-life in the body and can protect their internal materials and contents from enzymatic digestion. Moreover, they can be modified based on the intended profile of the target cell [34]. Exosomes offer several advantages over cell-based therapies, including low immunogenicity, no toxicity upon administration, ease of isolation, manipulation, and storage, and the ability to serve as carriers for therapeutic agents. Importantly, they also pose no risk of tumor formation and are free from the ethical concerns associated with stem cell transplantation [35,36]. MSC-Exo cell-free therapy has been reported to be effective in treating a wide range of pathological conditions, including graft-versus-host disease (GvHD) [37], inflammatory bowel disease [33], neurological disorders [38,39,40,41], diabetic wounds [42], liver diseases [43], COVID-19 [44,45,46,47], and others. Moreover, preclinical studies have shown promising results on the efficacy of MSC-Exo in the treatment of tumor diseases and demonstrated their potential in drug delivery [48,49]. Immunomodulatory properties of MSC-Exo have been demonstrated in experimental models of inflammatory and autoimmune diseases, including atopic dermatitis [50,51], systemic lupus erythematosus [52], and psoriasis [10,53,54,55].

Our aim was to compare the therapeutic effects of human umbilical cord blood-derived MSCs (hUCB-MSCs) and their exosomes (hUCB-MSC-Exo), preconditioned with different combinations of proinflammatory cytokines IL-17, IL-22, and TNF-a, whose levels are elevated in psoriasis, using an in vivo imiquimod (IMQ)-induced psoriasis-like skin inflammation model in mice.

## 2. Materials and Methods

### 2.1. Isolation and Cultivation of hUCB-MSCs

Umbilical cord blood (UCB) samples were collected from the umbilical vein immediately after birth with the informed consent of the mother, approved at the 4th meeting of the Local Ethics Commission (LEC) of the National Center for Biotechnology (NCB) (3 December 2021). The supernatant was carefully collected, and mononuclear cells were obtained by Ficoll density gradient centrifugation at 2500 rpm for 20 min. hUCB-MSC were washed twice with phosphate-buffered saline (PBS) and seeded at a density of 2 × 10^5^ cells/cm^2^ on Petri dishes in alpha-MEM medium (α-MEM) (Gibco^TM^, Life Technologies, Carlsbad, CA, USA) containing 10% fetal bovine serum (FBS) (Gibco™, Thermo Fisher Scientific, São Paulo, Brazil). After three days of hUCB-MSCs cultivation, non-adherent cells were removed, and by the end of the first week, the first colonies acquiring a spindle-shaped morphology were observed [56]. hUCB-MSCs were cultured until reaching 70% confluence and a density of 2 × 10^6^ cells/mL to establish a cell bank for further research. hUCB-MSCs were identified and characterized by flow cytometry and differentiation assays.

### 2.2. Identification of hUCB-MSCs by Flow Cytometry

Identification of isolated hUCB-MSCs was performed using the BD Stemflow™ Human MSC Analysis Kit (BD Biosciences, San Jose, CA, USA) according to the manufacturer’s instructions, on a Cytek Northern Lights™ flow cytometer (Cytek Biosciences, Inc., Fremont, CA, USA) equipped with SpectroFlo® software version 3.3.0.

### 2.3. Differentiation of hUCB-MSCs

For osteogenic differentiation of hUCB-MSCs, an induction medium (DMEM) containing 10^−7^ M dexamethasone, 10 mM β-glycerol phosphate, and 50 μM ascorbate-2-phosphate was used. The cells were cultured for 3 weeks, after which they were stained with Alizarin red S.

Differentiation into adipocytes was performed by culturing hUCB-MSCs in DMEM induction medium containing 10^−6^ M dexamethasone, 0.5 μM 3-isobutyl-1-methylxanthine, and 10 ng/mL insulin for 3 weeks. At the end of the cultivation, the cells were stained with Oil red O [57].

### 2.4. Preconditioning of hUCB-MSCs with Cytokines

Primary hUCB-MSC cultures were preconditioned with proinflammatory cytokines, 10 ng/mL TNF-α (ab259410), 50 ng/mL IL-17A (ab282392), and 10 ng/mL IL-22 (ab280331), individually and/or in combination for 24 h. All of these proteins were purchased from Abcam (Abcam Limited, Cambridge, UK).

hUCB-MSCs were divided into the following groups:

- Control (non-preconditioned MSCs);
- MSC preconditioned with TNF-α cytokine;
- MSC preconditioned with IL-22 cytokine;
- MSC preconditioned with IL-17 cytokine;
- MSC preconditioned with cytokines IL-22+IL-17;
- MSC preconditioned with cytokines TNF-α+IL-22+IL-17;
- MSC preconditioned with cytokines IL-22+TNF-α;
- MSC preconditioned with cytokines TNF-α+IL-17.

The choice of TNF-α, IL-17A, and IL-22 for preconditioning hUCB-MSCs is due to the fact that these cytokines play a key role in the development of psoriasis [58,59].

### 2.5. Isolation of Exosomes from Intact and Preconditioned hUCB-MSCs

After preconditioning hUCB-MSC cultures with proinflammatory cytokines, 24 mL of conditioned medium (8 mL per dish) was collected from each group of intact or preconditioned hUCB-MSCs. hUCB-MSC exosomes (hUCB-MSC-Exo) were isolated from conditioned medium by differential ultracentrifugation, as described by Gupta et al., using an Optima XPN-90 ultracentrifuge (Beckman Coulter, Inc., Brea, CA, USA) [60].

### 2.6. Size Determination of hUCB-MSC-Exo

The size of hUCB-MSC-Exo was determined using a NanoBrook 90Plus Zeta analyzer (Brookhaven Instruments, Holtsville, NY, USA). Briefly, a 50 μL hUCB-MSC-Exo sample was diluted in UltraPure water to a final volume of 5 mL at room temperature. The solution was filtered through a 0.45 μm filter to remove aggregates. A 4 mL cuvette was placed into the analyzer, and measurements were conducted at 25 °C with three runs of 15 min each at a scattering angle of 90 °C.

### 2.7. Measurement of the Zeta Potential of hUCB-MSC-Exo

The zeta potential of hUCB-MSC-Exo was measured using a NanoBrook 90Plus Zeta (Brookhaven Instruments, Holtsville, NY, USA) instrument with a BI-SREL electrode. Briefly, 50 µL of the hUCB-MSC-Exo sample was diluted in sterile, room-temperature PBS to a final volume of 3 mL. The solution was filtered through a 0.22 µm syringe filter and then transferred into a cuvette, where the electrode was immersed. The cuvette with the electrode was placed into the instrument, and measurements were performed at 25 °C.

### 2.8. Scanning Electron Microscopy (SEM) Characterization of hUCB-MSC-Exo

Scanning electron microscopy (SEM) characterization was performed using a Carl Zeiss Crossbeam 540 microscope (Carl Zeiss AG, Oberkochen, Germany). A drop of hUCB-MSC-Exo suspension diluted in PBS was placed on a clean glass coverslip and air-dried. After complete drying, the sample was coated with a 15 nm gold layer using a sputter coater. The morphology and size of the exosomes were analyzed based on the acquired high-resolution images.

### 2.9. Western Blot

Phenotypic profiles of hUCB-MSC-Exo were assessed by Western blot analysis (Figure S1). hUCB-MSC-Exo proteins were detected on GVS NitroBind transfer membranes (1215477, GVS North America, Sanford, ME, USA) using the anti-CD9 antibody [EPR23105-125] (ab263019, Abcam, Cambridge, UK) conjugated to rabbit-specific HRP secondary antibody. The results of the analysis were compared with a protein ladder Spectra^TM^ Multicolor Broad Range Protein Ladder (26634, Thermo Fischer Scientific, Waltham, MA, USA). Briefly, hUCB-MSC-Exo was lysed in RIPA buffer with sonication and then incubated on ice for 15 min, before mixing with a Laemmli sample buffer and heating at 70 °C for 10 min. Proteins were resolved on 12% SDS-PAGE gels and transferred to the nitrocellulose membrane. The membranes were blocked with 5% skim milk in TBST (Tris-buffered saline with 0.1% Tween 20) and incubated with primary antibody overnight at 4 °C with gentle agitation. Membranes were washed three times in TBST prior to treatment with secondary antibody for 1 h at room temperature with agitation. After washing three times in TBST, the ECL mixture was added to the membranes and visualized using an X-ray film cassette.

### 2.10. ELISA

After preconditioning hUCB-MSCs with cytokines, cell culture supernatants were collected. The collected cell culture supernatants from each group were analyzed for the level of secretion of PGE2 (ab133055, Abcam, UK), TGF-β1 (ab108912, Abcam, UK), and IL-6 (555220, BD Biosciences, USA) according to the manufacturer’s instructions. For the analysis, 96-well flat-bottom plates were used. The results were analyzed using a Thermo Scientific^TM^ Multiskan SkyHigh microplate spectrophotometer (ThermoFisher Scientific, Waltham, MA, USA).

### 2.11. RNA Extraction, cDNA Synthesis and Quantitative Real-Time PCR

hUCB-MSCs from the eight experimental conditions were collected by detaching them from the culture flasks using TrypLE^TM^ Express (Gibco, USA). Then, cells were pelleted by centrifugation at 300× *g* for 5 min. After centrifugation, the supernatant was discarded, and RNA isolation was initiated. First, each cell pellet was resuspended in 1 mL of PureZOL RNA Isolation Reagent (cat. 732-6890, Bio-Rad, Hercules, CA, USA). The samples were incubated at room temperature (RT) for 5 min, and then 0.2 mL of chloroform (Sigma-Aldrich, Gillingham, UK) was added to each sample. After some shaking, samples were incubated at RT. Following incubation, the tubes were centrifuged at 12,000× *g* for 15 min at 4 °C, resulting in three phases: a lower red organic phase, an interphase containing DNA, and an upper aqueous phase containing RNA. The aqueous phase containing RNA was carefully transferred to new RNase-free tubes without disturbing the interphase. To precipitate the RNA, 0.5 mL of isopropanol (Sisco Research Laboratories Pvt. Ltd., Mumbai, India) was added to each tube, and the contents were mixed by gently inverting the tubes several times. The tubes were incubated at RT for 10 min and then centrifuged at 12,000× *g* for 10 min at 4 °C. The supernatant was removed, and the RNA pellet was washed with 1 mL of 75% ethanol. The tubes were vortexed and centrifuged at 7500× *g* for 5 min at 4 °C. The supernatant was carefully removed, and the RNA pellets were air-dried at RT. The dried RNA pellets were resuspended in 50 µL of UltraPure^TM^ DNase/RNase-Free Distilled Water (Invitrogen^TM^, Carlsbad, CA, USA). The RNA was stored at −70 °C. cDNA (from all 8 groups of preconditioned hUCB-MSCs) synthesis was performed using the High-Capacity cDNA Reverse Transcription Kit (Applied Biosystems^TM^, Waltham, MA, USA). For each RNA sample, a reaction mixture was prepared with 10× RT (Real-Time) Buffer, 25× dNTP Mix (100 mM), 10× RT Random Primers, MultiScribe^TM^ Reverse Transcriptase, and RNase-free water. The total reaction volume was 10 µL, to which 10 µL of RNA was added, resulting in a total reaction volume of 20 µL. cDNA synthesis was carried out in a T100^TM^ Thermal Cycler (Bio-Rad Laboratories, Hercules, CA, USA). After obtaining cDNA, gene expression was analyzed by Real-Time Polymerase Chain Reaction (RT-PCR) using master mixes prepared for each reaction (10×Taq buffer with KCl, primer mix (containing FAM, forward and reverse primers), cDNA (from each sample of preconditioned hUCB-MSCs), MgCl, dNTPs, Taq polymerase, and RNase-free water). The total volume of each RT-PCR reaction was 20 µL. RT-PCR was performed in a CFX96^TM^ Touch Real-Time PCR Detection System (Bio-Rad Laboratories, USA). The RT-PCR cycle program consisted of 40 cycles, and the data were analyzed based on the fluorescence signals recorded at the stage of reading the samples in each tube. The primers were prepared by the Laboratory for Development of Molecular Diagnostic Approaches of the NCB. The following primers were used for RT-PCR (Table 1).

### 2.12. Mice

BALB/c male mice were purchased from the Federal State Budgetary Scientific Institution “Federal Research Center Institute of Cytology Genetics of the Siberian Branch of the Russian Academy of Sciences”. The animals were quarantined for two weeks after their arrival. BALB/c male mice were housed in an environmentally controlled room with a 12:12 h light-dark cycle and free access to laboratory chow and water. Mice between 8 and 12 weeks of age were used. The protocol for mouse use was approved by the LEC of the NCB (No. 4 of 3 December 2021). All animal experiments were performed under isoflurane anesthesia.

### 2.13. Imiquimod (IMQ)-Induced Psoriasis-like Skin Inflammation in Mice

**Figure 1:**
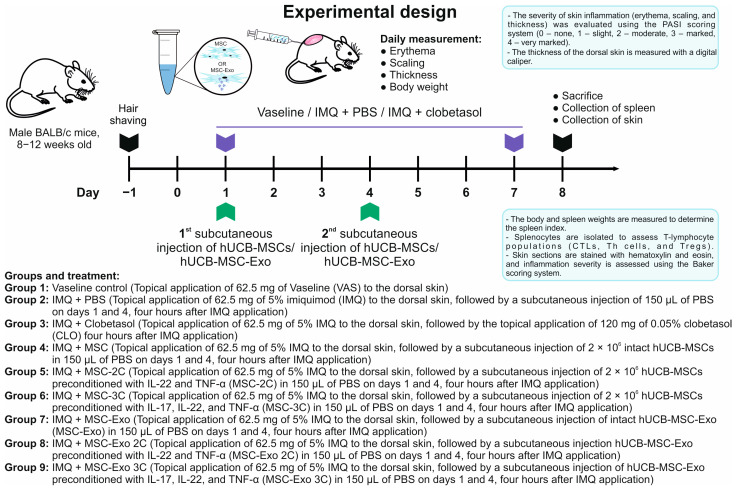
Experimental design and treatment groups.

The experimental unit is a cage of animals. The mouse model of psoriasis-like skin inflammation was induced as described by van der Fits et al. [61]. Briefly, 2 days before inducing skin inflammation, the dorsal skin of animals was shaved using a hair clipper, and residual hair was removed using Veet Minima depilatory cream (Reckitt Benckiser, Chartres Cedex, France). In total, 62.5 mg of Keravort cream (Glenmark Pharmaceuticals Ltd., Mumbai, India) containing a daily dose of 3.125 mg imiquimod (IMQ) was applied to the shaved dorsal skin of mice for 7 consecutive days to achieve optimal inflammation. Each sachet of cream contains 250 mg of Keravort cream, containing 12.5 mg of IMQ; therefore, the required single dose is a quarter of a sachet. IMQ was applied to the middle part of the midsagittal plane of the back of each animal. hUCB-MSCs or hUCB-MSC-Exo samples were subcutaneously injected into the dorsal skin on days 1 and 4, 4 h after IMQ application. hUCB-MSC samples were injected at a dose of 2 × 10^6^ cells in 150 μL of sterile PBS. Animals in the hUCB-MSC-Exo-treated groups received an equivalent volume of exosome suspension in PBS. The comparator drug was Derylife 0.05% cream (World Medicine İlaç Sanayi ve Ticaret A.Ş., Istanbul, Türkiye), containing the glucocorticosteroid clobetasol (CLO) as the active ingredient. It was applied daily to the dorsal skin for 7 days at a dose of 120 mg, 4 h after IMQ application. Each group contained 4 mice. The total number of animals in each experiment was 36. Two independent experiments were conducted. For each analysis, all animals were taken into account. The mice were euthanized on day 8 for analysis [62]. A schematic diagram representing the experimental design is shown in Figure 1.

### 2.14. Scoring of Psoriasis Severity

To score the severity of inflammation in the dorsal skin, the Psoriasis Area and Severity Index (PASI) scoring system was used as described by van der Fits et al. [61]. Erythema, scaling, and thickness were scored independently on a scale from 0 to 4 (0—none, 1—slight, 2—moderate, 3—marked, and 4—very marked). The combination of the above independent scores is considered a cumulative score for the PASI, which serves as a measure of psoriasis severity. Dorsal skin thickness was measured using a Matrix 31611 digital caliper (Matrix, Suzhou, China). Body weight was recorded daily [63,64].

### 2.15. Evaluation of the Systemic Effect of IMQ on the Spleen

After 24 h of the final application of IMQ, the animals were euthanized by carbon dioxide (CO_2_). The spleen was excised and weighed to calculate the spleen index using the following formula [62]:

$$Spleen\;index=\frac{Spleen\;weight}{Body\;weight}$$

### 2.16. Flow Cytometry

Splenocyte isolation was performed according to the protocol described by Grosjean et al. [65]. Splenocyte staining with monoclonal antibodies and subsequent flow cytometric analysis were performed on a Cytek NorthernLights^TM^ flow cytometer (Cytek Biosciences, Inc., Fremont, CA, USA) with SpectroFlo^®^ software. The following antibodies were used: CD4 conjugated to Super Bright™ 702; CD8A conjugated to PerCP; and CD25 conjugated to PE. All of these antibodies were purchased from eBioscience or BD Biosciences. Cell suspension and washing were performed using StainBuffer (FBS) (BD Biosciences). Live and dead cell populations were determined using a Live and Dead Cell Assay Kit (Calcein AM, 7-AAD) (ab270789, Abcam).

### 2.17. Histopathology Studies

Dorsal skin samples were collected, fixed in 10% formalin, embedded in paraffin, sectioned, and stained with hematoxylin and eosin (H&E). The prepared slides were examined under an AxioScope.A1 microscope (Carl Zeiss Microscopy GmbH, Göttingen, Germany) at magnifications of 200× and 400×. Images of the sections were taken using ZEN 3.1 software (Blue Edition) and an AxioCam MRc5 camera (Carl Zeiss Microscopy GmbH, Göttingen, Germany). The final image scales of the sections were 785× and 1570×. The obtained images were processed using ImageJ software version 1.54g (Bethesda, MD, USA).

The sections were scored blindly for psoriasiform features on a scale ranging from 0 to 10 (Table 2) and compared with intact animal skin samples [66,67].

### 2.18. Statistical Analysis

The data were expressed as means ± standard deviations (SDs). Statistical comparisons between two groups were performed using an unpaired two-tailed Student’s *t*-test. The figures were generated using GraphPad Prism 8.0.1 (GraphPad Software, Inc., San Diego, CA, USA). The *p*-value was considered significant at * *p* < 0.05, ** *p* < 0.01, and *** *p* < 0.001, **** *p* < 0.0001. The pictures for the article were created in the program CorelDRAW^®^ X7 version 17.3.0.772 (Corel Corporation, Ottawa, ON, Canada).

## 3. Results

### 3.1. Identification of hUCB-MSCs by Flow Cytometry

**Figure 2:**
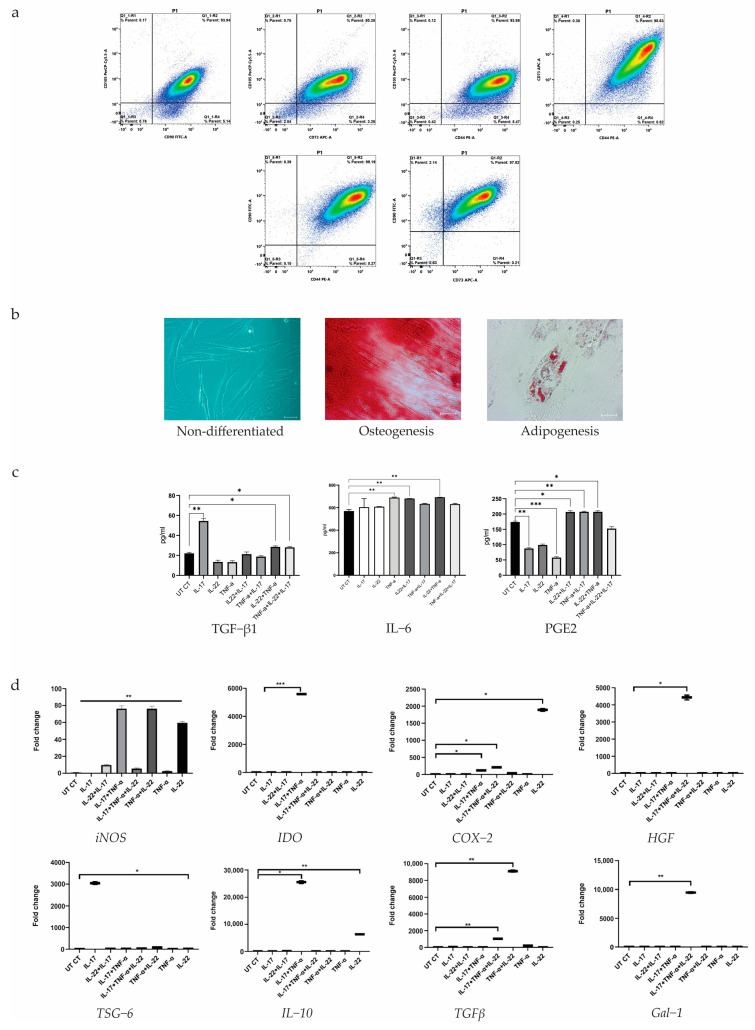
Immunomodulatory and immunosuppressive properties of hUCB-MSCs. (**a**) Identification of hUCB-MSCs by flow cytometry to determine surface expression of CD73, CD90, CD105, and CD44. (**b**) Non-differentiated hUCB-MSCs. The differentiation potential of hUCB-MSCs into osteoblasts and adipocytes. Scale bar: 50 μm. (**c**) ELISA analysis of PGE2, TGF-β1, and IL-6 secretion in cell culture supernatants demonstrated the immunomodulatory and immunosuppressive properties of hUCB-MSCs following preconditioning with psoriasis-associated proinflammatory cytokines TNF-α, IL-17A, and IL-22. (**d**) The results of real-time PCR analysis of immunosuppressive gene expression in hUCB-MSCs preconditioned with cytokines. * *p* < 0.05; ** *p* < 0.01; *** *p* < 0.001.

Identification of hUCB-MSCs was performed by flow cytometry using a panel of cell surface markers containing antibodies against CD73, CD90, CD105, and CD44 (Figure 2a). The results of the analysis showed that cells isolated from human umbilical cord blood have the MSC phenotype with reliable expression of all the listed cell surface markers.

### 3.2. Differentiation of hUCB-MSCs

To demonstrate the differentiation potential of the isolated hUCB-MSC cultures, we induced their differentiation into osteoblasts and adipocytes using specific differentiation media (Figure 2b). The differentiation ability of hUCB-MSCs indicates their multipotency, demonstrating their potential to differentiate into osteoblasts and adipocytes.

### 3.3. Analysis of the Production of Immunomodulatory and Immunosuppressive Mediators by hUCB-MSCs Using ELISA

ELISA analysis of the conditioned medium allowed us to determine the effect of cytokines and their various combinations on the immunomodulatory and immunosuppressive mediators of hUCB-MSCs (Figure 2c). Secretion of TGF-β1 was observed in all groups of preconditioned hUCB-MSCs. The secretion level of TGF-β1 remained unchanged in intact hUCB-MSCs and in hUCB-MSC groups preconditioned with the cytokine combinations IL-22+IL-17 and TNF-α+IL-17. In the groups of hUCB-MSCs preconditioned with combinations of cytokines IL-22+TNF-α and TNF-α+IL-22+IL-17, the secretion level of TGF-β1 factor was higher compared to the control. Interestingly, in the group of hUCB-MSCs preconditioned with the cytokine IL-17 alone, the secretion of TGF-β1 was two-fold higher compared to the control. Secretion of the cytokine IL-6 was observed in all groups of preconditioned hUCB-MSCs. hUCB-MSCs preconditioned with TNF-α alone and the cytokine combinations IL-22+IL-17 and IL-22+TNF-α showed higher levels of IL-6 cytokine secretion compared to controls. PGE2 secretion was observed in all groups of preconditioned hUCB-MSCs. Preconditioning of hUCB-MSCs with cytokine combinations IL-22+IL-17, TNF-α+IL-17 and IL-22+TNF-α increased PGE2 protein secretion levels compared to the control. ELISA results showed that the most significant immunomodulatory and immunosuppressive properties of hUCB-MSCs were observed in groups preconditioned with cytokine combinations.

### 3.4. Analysis of the Expression of Immunosuppressive, Immunomodulatory and Immunoregulatory Genes of hUCB-MSCs by Real-Time PCR

To determine the expression levels of immunosuppressive genes in hUCB-MSCs, RT-PCR was performed (Figure 2d). It was found that the expression level of the inducible nitric oxide synthase (*iNOS*) was slightly increased in hUCB-MSCs preconditioned with the cytokine combinations IL-22+IL-17 and IL-17+TNF-α+IL-22, as well as with TNF-α only, compared to the control. On the other hand, the *iNOS* gene expression level was significantly increased in hUCB-MSCs preconditioned with the cytokine combinations IL-17+TNF-α and TNF-α+IL-22, and with the cytokine IL-22 alone, compared to the control. The indoleamine 2,3-dioxygenase (*IDO*) gene expression level was significantly increased in hUCB-MSCs preconditioned with the cytokine combination IL-17+TNF-α compared to the control. In all other groups, the level of *IDO* gene expression was at the same level as in the control sample. The expression level of cyclooxygenase-2 (*COX-2*) was slightly increased in hUCB-MSCs preconditioned with the cytokine combinations IL-17+TNF-α and IL-17+TNF-α+IL-22 compared to the control. In the hUCB-MSC group preconditioned with IL-22 alone, a significant increase in *COX-2* gene expression was observed compared to the control. In the remaining hUCB-MSC samples, *COX-2* gene expression was not observed. The expression level of the hepatocyte growth factor (*HGF*) gene increased significantly only in hUCB-MSCs preconditioned with the combination of cytokines IL-17+TNF-α+IL-22 compared to the control. However, in the remaining hUCB-MSC samples, preconditioned with cytokines separately or in other combinations, expression of the *HGF* gene was not observed. The expression level of tumor necrosis factor-inducible gene 6 (*TSG-6*) was slightly increased in hUCB-MSCs preconditioned with the combination of cytokines TNF-α+IL-22 compared to the control. A significant increase in the expression level of *TSG-6* was observed in hUCB-MSCs preconditioned with IL-17 alone compared to the control. Analysis of cytokine interleukin-10 (*IL-10*) gene expression showed a slight increase in expression in the group of hUCB-MSC preconditioned with IL-22 only, and a significant increase in the combination of cytokines IL-17+TNF-α compared to the control. The expression level of the transforming growth factor beta (*TGFβ*) gene was slightly increased in the groups IL-17, TNF-α, and IL-17+TNF-α+IL-22 compared to the control. However, a significant increase in the level of *TGFβ* gene expression was observed in the hUCB-MSC group preconditioned with the combination of cytokines TNF-α+IL-22 compared to the control. The expression level of the galectin 1 (*Gal-1*) gene was significantly increased only in the group of hUCB-MSCs preconditioned with the combination of cytokines IL-17+TNF-α+IL-22 compared to the control. In the remaining groups, *Gal-1* gene expression was not observed.

### 3.5. Isolation of Exosomes from hUCB-MSCs (hUCB-MSC-Exo)

**Figure 3:**
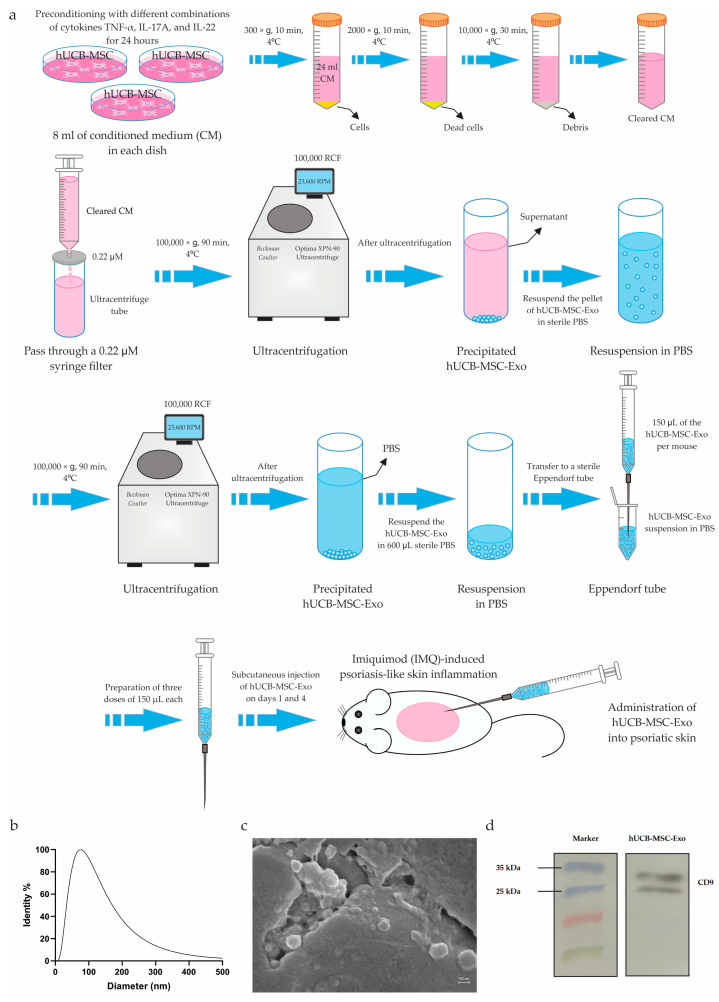
Isolation and characterization of hUCB-MSC-Exo. (**a**) Protocol for isolating exosomes from the hUCB-MSCs culture medium using the differential ultracentrifugation technique. (**b**) Nanoparticle size analysis revealed that hUCB-MSC-Exo had an average diameter of 85.34 nm, which falls within the characteristic size range of exosomes (30–200 nm). (**c**) SEM image of hUCB-MSC-Exo showing spherical morphology and size consistent with exosomes. Scale bar = 100 nm. (**d**) Western blot analysis confirmed the presence of CD9 protein on the surface of hUCB-MSC-Exo, which is one of the established exosomal markers.

hUCB-MSC-derived exosomes (hUCB-MSC-Exo) were isolated using the differential centrifugation technique, as shown in Figure 3a. Characterization of hUCB-MSC-Exo was confirmed by nanoparticle size and zeta potential measurements, SEM analysis, and detection of the protein marker CD9 (Figure 3b–d). The average diameter of hUCB-MSC-Exo was 85.34 nm, as determined by the NanoBrook 90Plus Zeta analyzer, and 95.93 nm according to SEM analysis, both of which fall within the characteristic size range of exosomes (30–200 nm). The surface charge of hUCB-MSC-Exo was maintained relatively constant (between −21.24 and −25.16 mV), indicating stable dispersion in solution and suggesting efficient interactions with target cell membranes due to their negative charge.

### 3.6. The Injection of hUCB-MSCs and hUCB-MSC-Exo Attenuated Both the Development and Severity of Psoriasis in Mice

**Figure 4:**
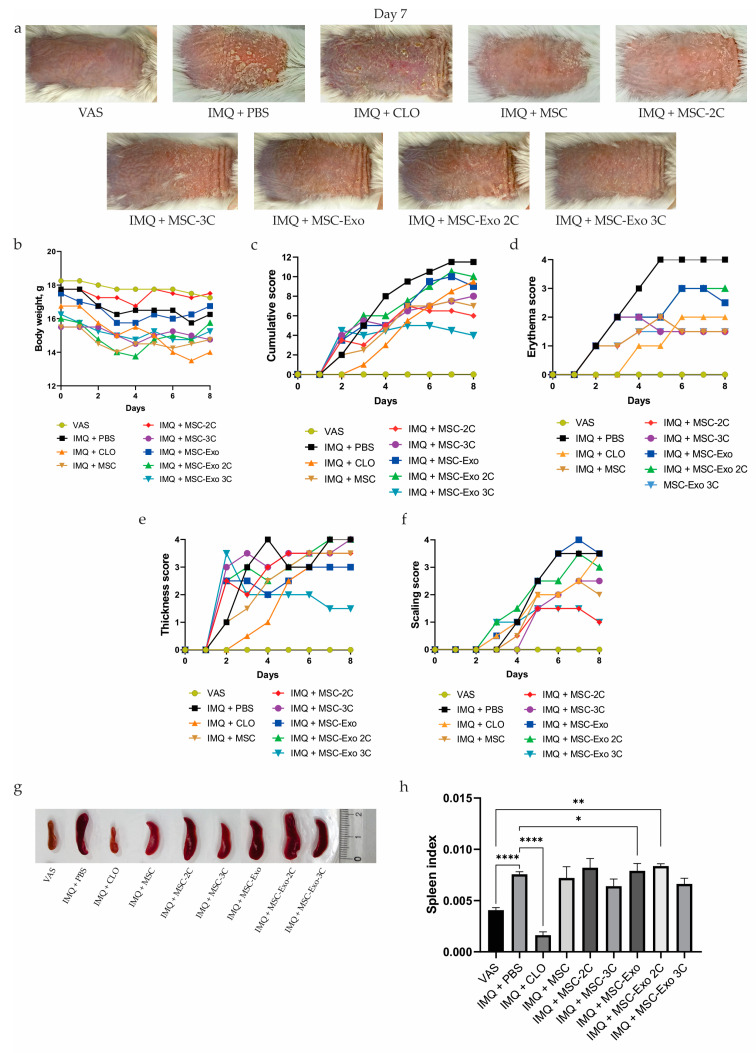
IMQ + MSC, IMQ + MSC-2C, and IMQ + MSC-Exo 3C significantly ameliorated psoriatic symptoms in IMQ-induced mice. (**a**) Representative photographs of mouse dorsal skin after treatment on day 7. (**b**) The body weight of mice was assessed daily. (**c**) A cumulative score assessing the combination of three signs of psoriasis (erythema, scaling, and thickness) was scored daily. (**d**–**f**) Different levels of erythema, scaling, and thickness of dorsal skin were scored daily. (**g**) Representative photographs of spleens on day 8. (**h**) A slight decrease in the spleen index was observed in the IMQ + MSC-3C and IMQ + MSC-Exo 3C groups compared to the IMQ + PBS group. * *p* < 0.05; ** *p* < 0.01; **** *p* < 0.0001.

To better understand the role of hUCB-MSCs and hUCB-MSC-Exo injection in psoriasis pathogenesis, IMQ was topically applied to the dorsal skin of mice daily for 7 consecutive days (Figure 1). The severity of psoriasis-like skin inflammation was assessed daily using the PASI scoring system. In the intact group that received only topical therapy with Vaseline (VAS) at a dose of 62.5 mg, signs of psoriasis-like inflammation were not observed throughout the entire period of the experiment (Figure 4a). Treatment of the skin with VAS did not affect the dynamics of body weight gain in experimental animals, which indicates the safety of this medication (Figure 4b). In the control group (IMQ + PBS), which received phosphate-buffered saline (PBS) injections on days 1 and 4, PASI scores began to increase rapidly from the second day of IMQ application and reached their peak by day 7 of the experiment. Phenotypically, the dorsal skin of the mice displayed typical symptoms of erythema, scaling, and thickening, followed by inflammation, which progressively worsened until the end of IMQ application on day 7 (Figure 4a). The PASI score also continued to rise. By the end of the experiment, the cumulative score was 11.5 (Figure 4c). These results indicate the success of reproducing the disease model. Treatment with the glucocorticosteroid drug clobetasol (IMQ + CLO) was effective only at the initial stage of the experiment, up to four days after the start of IMQ application. Then, from day 4 until the end of the experiment, all PASI parameters (erythema, scaling, and thickness) tended to worsen (Figure 4d–f).

By the end of the experiment, the cumulative score in the IMQ + CLO group was 9.5, which is only 17.4% lower than the PBS score (Figure 4c). Such a high PASI rate in the IMQ + CLO group may be associated with the immunosuppressive effect of steroid medications when taken for a long time, which significantly reduces their therapeutic effectiveness. It should also be noted that the mice in the IMQ + CLO group showed a steady tendency to lose body weight during the experiment, which indicates the adverse effects of long-term use of steroid medications (Figure 4b).

In the groups treated with intact hUCB-MSCs (IMQ + MSC) and hUCB-MSCs preconditioned with combinations of cytokines IL-22+TNF-α (IMQ + MSC-2C) and IL-17+IL-22+TNF-α (IMQ + MSC-3C), the following clinical picture was observed. Psoriasis symptoms first appeared in all groups on the second day of IMQ application. However, the significant skin thickening observed on the second day may not result from the accelerated development of IMQ-induced inflammatory symptoms but rather from the subcutaneous injection of IMQ + MSC (Figure 4f). Characteristic psoriasis symptoms began to appear in these groups on the third day of IMQ application (Figure 4d–f). The best PASI score across all measured parameters among the groups treated with MSCs was observed in the IMQ + MSC-2C group. On the eighth day of the experiment (before euthanasia), the cumulative score in this group was 6, which was 47.5% lower than in the IMQ + PBS group (11.5). For comparison, the cumulative PASI scores in the IMQ + MSC and IMQ + MSC-3C groups were 7 and 8, respectively (Figure 4c). By day 7, mice treated with IMQ + MSC and IMQ + MSC-2C exhibited a significant reduction in psoriasis-like inflammation symptoms, characterized by decreased erythema and scaling areas, as well as reduced skin thickness (Figure 4a). Moreover, during the experiment, significant fluctuations in the body weight of mice were not recorded in the IMQ + MSC, IMQ + MSC-2C, and IMQ + MSC-3C groups (Figure 4b).

In the groups treated with exosomes isolated from intact hUCB-MSCs (MSC-Exo) and hUCB-MSCs preconditioned with combinations of cytokines IL-22+TNF-α (IMQ + MSC-Exo 2C) and IL-17+IL-22+TNF-α (IMQ + MSC-Exo 3C), the following clinical picture was observed. The onset of disease symptoms in all groups was observed starting from the second day of IMQ application (Figure 4d–f). As in the IMQ + MSC, IMQ + MSC-2C, and IMQ + MSC-3C groups, significant skin thickening was observed on the second day after the first subcutaneous injection of exosomes, particularly at the injection site (Figure 4f). Starting on the third day of IMQ application, characteristic psoriasis symptoms appeared in all groups receiving exosome therapy. However, in the IMQ + MSC-Exo 3C group, the cumulative PASI score increased only until the fourth day, reaching 4.5 (Figure 3c). Furthermore, all recorded rates in this group remained at a consistently low level until the end of the experiment (Figure 4c). On the eighth day of the experiment, the cumulative score in the IMQ + MSC-Exo 3C group was 4, which was 65.2% lower than in the IMQ + PBS group (11.5) (Figure 4c). It was noted that there was significant improvement in the skin, in particular a decrease in erythema, scaling, and thickness (Figure 4a).

After euthanasia, the spleens of the mice were excised and their size and weight were measured (Figure 4g). Next, the spleen index was calculated for all experimental groups (Figure 4h). Calculations of the spleen index revealed no significant statistical differences between the study groups. Among the groups treated with hUCB-MSCs or hUCB-MSC-Exo, only the IMQ + MSC-3C (0.0064) and IMQ + MSC-Exo 3C (0.0066) groups showed a slight decrease in spleen index compared to the IMQ + PBS group (0.0076) by 15.79% and 13.16%, respectively. However, in the IMQ + CLO group, a significant decrease in spleen weight was observed, even in comparison with the VAS group, which may be evidence of suppression of the immune system by long-term steroid therapy. The spleen index in the IMQ + CLO group was 0.0016, which is 60.98% less than in the VAS group (0.0041) (Figure 4h).

### 3.7. Evaluation of T-Lymphocyte Populations by Flow Cytometry

**Figure 5:**
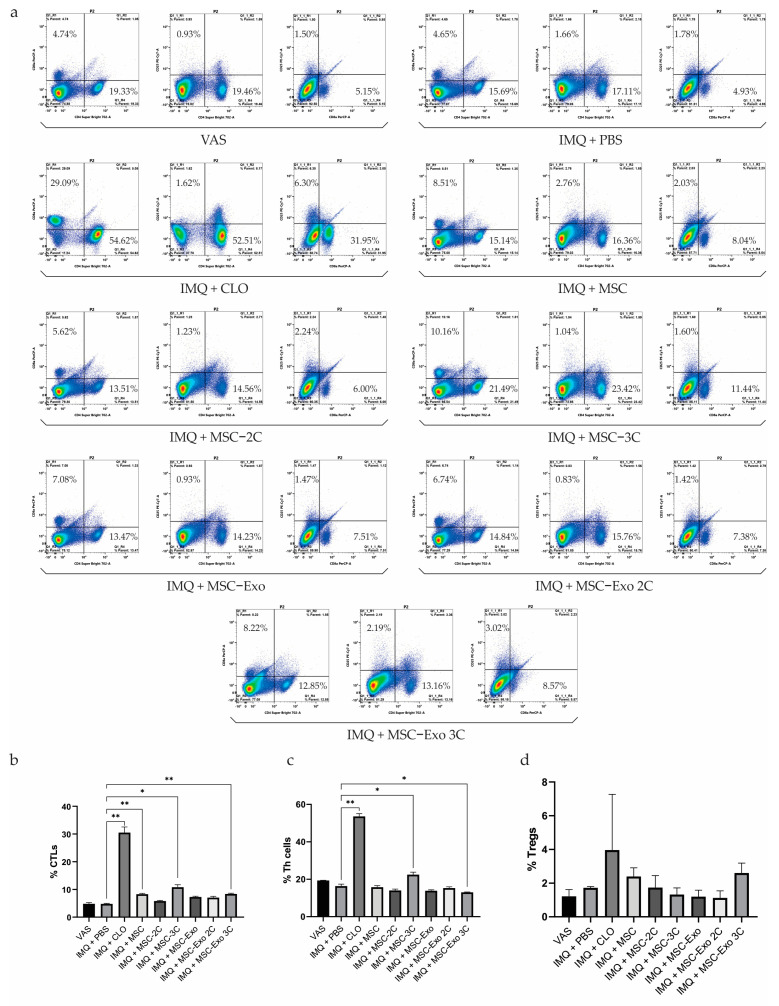
Flow cytometric evaluation of T-lymphocyte populations in the mouse spleen. (**a**) Representative flow cytometry staining of CD8+ CTLs, CD4+ Th cells, and CD25+ Tregs from the mouse spleen. (**b**–**d**) Quantification of the percentage of CD8+ CTLs, CD4+ Th cells, and CD25+ Tregs from flow cytometry data. * *p* < 0.05; ** *p* < 0.01.

Flow cytometry of spleen cells from mice treated with intact or cytokine-preconditioned hUCB-MSCs and their exosomes allowed us to assess the expression levels of T-lymphocyte subsets, including cytotoxic T-lymphocytes (CTLs), helper T-lymphocytes (Th), and regulatory T-lymphocytes (Tregs). The results of flow cytometry are presented in Figure 5a.

Assessment of the populations of CD8+ CTLs, CD4+ Th cells, and CD25+ Tregs by flow cytometry showed the following results (Figure 5b–d). There was an increase in the number of CTLs in all groups receiving both treatment with CLO and therapy based on hUCB-MSCs and their exosomes. In particular, a statistically significant increase in CTLs compared to the IMQ + PBS group (4.8%) was found in the IMQ + CLO, IMQ + MSC, IMQ + MSC 3C, and IMQ + MSC-Exo 3C groups—6.4 times (30.5%), 1.7 times (8.3%), 2.3 times (10.8%), and 1.8 times (8.4%), respectively. At the same time, among all treated groups, the greatest decrease in the percentage of CTLs was observed in the IMQ + MSC-2C group (5.81%); however, this value was still 1.2 times higher than in the IMQ + PBS group (4.8%) (Figure 5b). Evaluation of Th cell populations revealed a 1.2-fold decrease in Th cell numbers in the IMQ + PBS group (16.4%) compared to the VAS group (19.4%). An increase in the number of Th cells compared to the IMQ + PBS group was observed in the IMQ + CLO and IMQ + MSC 3C groups by 3.3 times (53.6%) and 1.4 times (22.5%), respectively. On the contrary, compared to the IMQ + PBS group, a slight decrease in Th cells was recorded in the IMQ + MSC (15.8%) and IMQ + MSC-Exo 2C (14.0%) groups, as well as in all groups treated with exosomes. At the same time, the greatest decrease in the number of Th cells was found in the MSC-Exo 3C group—1.3 times (13.0%) compared to the IMQ + PBS group (Figure 5c). Analysis of Treg populations revealed a 1.4-fold increase in the number of cells in the IMQ + PBS group (1.7%) compared to the VAS group (1.2%). Compared to the IMQ + PBS group, an increase in the percentage of Tregs was observed in the following groups: IMQ + CLO by 2.3 times (3.9%), IMQ + MSC by 1.4 times (2.4%), and IMQ + MSC-Exo 3C by 1.5 times (2.6%) (Figure 5d).

### 3.8. hUCB-MSCs and hUCB-MSC-Exo Alleviate Psoriasis Symptoms According to Histological Examination

**Figure 6:**
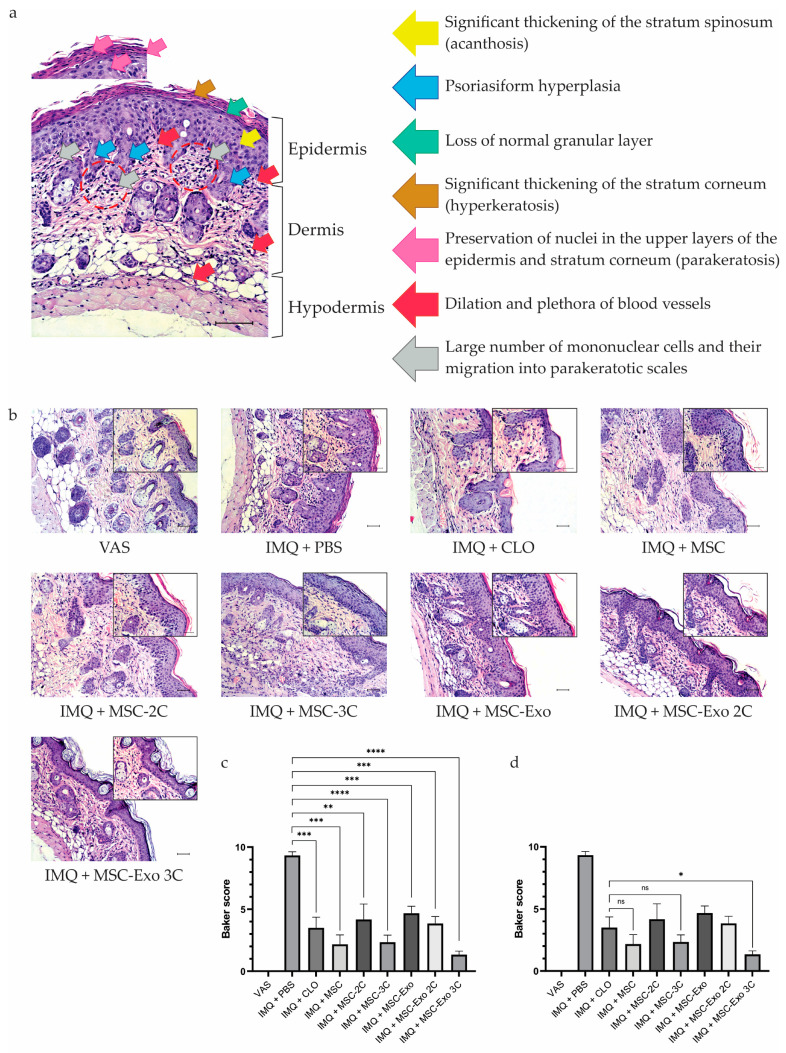
Histopathological analysis of dorsal skin samples showed that IMQ + MSC, IMQ + MSC-3C, and IMQ + MSC-Exo 3C significantly suppressed the IMQ-induced inflammatory response. (**a**) Histopathological analysis of a mouse dorsal skin sample with IMQ-induced inflammation (IMQ + PBS group). Scale bar: 100 μm. (**b**) Representative H&E staining of skin samples from treated mice. Scale bar: 100 μm. (**c**) Total histopathological assessment of inflammation severity according to the Baker scoring system. Statistical comparisons were performed against the untreated control group (IMQ + PBS). (**d**) Additional analysis of inflammation severity comparing IMQ + MSC, IMQ + MSC-3C, and IMQ + MSC-Exo-3C groups with the reference treatment group (IMQ + CLO) according to the Baker scoring system. Data are presented as means ± SDs. * *p* < 0.05, ** *p* < 0.01, *** *p* < 0.001, **** *p* < 0.0001, ns—not significant.

Histological examination revealed that in the IMQ + PBS group, the skin of mice was characterized by a significant thickening of the stratum spinosum (acanthosis) and epidermal “psoriasiform hyperplasia” with elongation of the rete ridges, thinning of the suprapapillary plates, and loss of granular layers, as well as a significant thickening of the stratum corneum (hyperkeratosis). Preservation of nuclei in the upper layers of the epidermis and stratum corneum (parakeratosis) was noted, as well as migration of inflammatory cells through the epidermis into parakeratotic scales (Munro microabscesses). Dilatation and congestion of blood vessels in the dermal layer were also noted (Figure 6a) [68].

Histological photographs of all treated groups are presented in Figure 6b. The IMQ + PBS group recorded the highest score according to the Baker scoring system (9.33) (Figure 6c). A decrease in the overall Baker score was observed across all groups, but the most significant decreases were observed in the groups IMQ + CLO—3.5; IMQ + MSC—2.2; IMQ + MSC-3C—2.3; and IMQ + MSC-Exo 3C—1.3, which were less than those in the IMQ + PBS group by 62.4%, 76.3%, 75.3%, and 86.3%, respectively (Figure 6c). Interestingly, the IMQ + MSC, IMQ + MSC-3C, and IMQ + MSC-Exo 3C groups showed Baker scores lower than even the IMQ + CLO group by 37.1%, 34.3%, and 62.9%, respectively, which is significantly superior to the results of the glucocorticosteroid medication clobetasol. Compared to the intact VAS group, the IMQ + MSC, IMQ + MSC-3C, and IMQ + MSC-Exo 3C groups showed reductions in epidermal thickness, hyperkeratosis, psoriasiform hyperplasia, and lymphocytic infiltration induced by IMQ therapy. However, the best results in the histopathological assessment were recorded in the IMQ + MSC-Exo 3C group, which correlated with the PASI results.

## 4. Discussion

In this study, we successfully isolated hUCB-MSCs, which demonstrated osteogenic and adipogenic differentiation capabilities. Additionally, we tested the isolated hUCB-MSCs for the expression of classical cell surface markers (CD90, CD73, CD105, and CD44) used to define MSCs [69]. Next, we analyzed the secretion levels of immunomodulatory and immunosuppressive mediators by hUCB-MSCs using ELISA. It is well known that psoriasis is a genetically determined immune-mediated inflammatory disease. Furthermore, inflammatory cells and their released products, such as cytokines, chemokines, and growth factors, eventually contribute to keratinocyte hyperproliferation, epidermal thickness, and angiogenesis, which results in prominent blood vessel ectasia [70]. Based on this, we measured the secretion levels of growth factors and cytokines, including TGF-β1, IL-6, and PGE2, in the conditioned medium after hUCB-MSCs preconditioning with IL-17, IL-22, TNF-α, and their combinations. TGF-β is a multipotent cytokine that controls cell proliferation and differentiation [71]. TGF-β1 targets the skin, with receptors found in epidermal keratinocytes [72]. Psoriatic patients have elevated levels of TGF-β1 in their epidermis and serum, which correlates with disease severity [73,74,75]. TGF-β1, 2, and 3 are involved in almost all aspects of MSC function [76]. In particular, TGF-β1 stimulates the migration of MSCs to damaged sites [77], regulates the activity of macrophages, and suppresses inflammation [78]. In our study, we observed that compared to the control, a higher level of TGF-β1 secretion was observed in groups of hUCB-MSCs preconditioned with combinations of cytokines IL-22+TNF-α (28.5 pg/mL) and TNF-α+IL-22+IL-17 (28.0 pg/mL), and more than two-fold when exposed to the cytokine IL-17 only (54.4 pg/mL) compared to the control (22.0 pg/mL). At the same time, a lower level of TGF-β1 secretion was observed in groups of hUCB-MSCs preconditioned with cytokines IL-22 (13.4 pg/mL) and TNF-α (13.2 pg/mL) separately compared to the control (22.0 pg/mL). It should be noted that TGF-β1 plays a controversial role in psoriasis. TGF-β1 has been shown to inhibit keratinocyte growth in psoriasis but may also enhance keratinocyte proliferation as a result of increased levels of inflammatory cytokines and chemokines (IL-1, IL-6, IL-8). TGF-β1 also increases fibroblast proliferation, induces angiogenesis, and causes vasodilation in early psoriasis [79,80,81]. Moreover, there is strong evidence that increased expression of latent TGF-β1 in the epidermis is associated with psoriasis-like skin inflammation [72]. Interleukin-6 (IL-6) is a universal cytokine that plays a crucial role in modulating the immune response, regulating inflammation and various physiological processes in the body. Its wide range of functions highlights its importance in maintaining health [82]. Dysregulation of IL-6 is closely associated with many diseases, including psoriasis [83]. IL-6 is found in the blood serum and skin lesions of patients with psoriasis [84]. IL-6, like IL-1 and TNF-α, is a proinflammatory cytokine produced by various cell types and has a wide range of biological effects [85]. In psoriasis, IL-6 is produced by keratinocytes, fibroblasts, endothelial cells, dendritic cells (DCs), macrophages, and T helper 17 cells. IL-6 has been shown to have numerous biological effects in affected tissues, including keratinocyte growth, activation, and production of proinflammatory cytokines/chemokines (especially in synergy with TNF-α and IL-17A); production of proinflammatory cytokines and chemokines by macrophages and DCs; differentiation of T helper 17 cells; expression of adhesion molecules on endothelial cells; and differentiation of neutrophils [83]. IL-6 is a potential marker of disease activity in patients with psoriasis [86]. In our study, we observed an increase in the level of IL-6 protein secretion in all groups of hUCB-MSCs preconditioned with either one of the cytokines or their combinations. At the same time, the highest levels of IL-6 secretion were observed in groups of hUCB-MSCs preconditioned with TNF-α alone (688.6 pg/mL), as well as with combinations of IL-22 + IL-17 (680.5 pg/mL) and IL-22 + TNF-α (691.9 pg/mL), compared to the control (569.6 pg/mL). It should be noted that MSCs secrete copious amounts of IL-6 protein [87]. As we can see, additional preconditioning of hUCB-MSCs with proinflammatory cytokines (IL-17, IL-22, and TNF-α), which are elevated in patients with psoriasis, promotes higher expression of IL-6 protein. IL-6 is involved in human MSC (hMSC)-induced immunosuppression. It was shown that cells in which IL-6 was suppressed exhibited a reduced ability to suppress the proliferation of activated T cells. Moreover, suppression of IL-6 significantly blocked the proliferation ability of hMSCs. It has also been shown that increasing intracellular IL-6 levels without restoring extracellular levels can restore the proliferative impairment observed in IL-6-suppressed hMSC [88]. Thus, preconditioning of hUCB-MSCs with cytokines IL-17, IL-22, and TNF-α, either individually or in combination, promotes increased expression of IL-6 protein and, accordingly, higher immunosuppressive activity of hUCB-MSCs. Prostaglandin E2 (PGE2) is a bioactive lipid that exerts a wide range of biological effects associated with inflammation and cancer. PGE2 has multiple effects on cell proliferation, apoptosis, angiogenesis, inflammation, and immune surveillance [89]. In MSC therapy, their ability to migrate to sites of damage plays a critical role. PGE2 is the major prostaglandin produced by cyclooxygenase (COX) enzymes and is involved in the inflammatory response. Evidence suggests that PGE2 may promote MSC migration [90]. The role of PGE2 in psoriasis is multifaceted [91]. PGE2 has been reported to shift adaptive immunity toward Th1 and Th17 responses by influencing DCs [92], stimulating the expansion of Th17 cells, and promoting the differentiation of Th1 cells [93]. In the present study, preconditioning of hUCB-MSCs with cytokine combinations IL-22+IL-17 (206.6 pg/mL), TNF-α+IL-17 (207.2 pg/mL), and IL-22+TNF-α (207.1 pg/mL) increased PGE2 protein secretion levels compared to the control (173.8 pg/mL). Based on the obtained results, an increase in PGE2 expression as a result of preconditioning MSCs with the indicated combinations of cytokines will lead to an increase in the migration capacity of MSC and, accordingly, an increase in therapeutic efficacy.

Subsequently, the following key genes of MSCs were analyzed for expression levels after preconditioning with proinflammatory cytokines IL-17, IL-22, and TNF-α: immunosuppressive genes *iNOS* and *IDO* [94], immunomodulatory genes *COX-2*, *TGFβ*, and *Gal-1* [78,95,96], *HGF* and *TSG-6* genes maintaining MSC stemness [97,98], and the gene *IL-10*, which is an immunoregulatory cytokine [99]. RT-PCR analysis of gene expression showed that preconditioning of hUCB-MSCs with IL-22 alone, as well as with combinations of IL-17+TNF-α and TNF-α+IL-22 cytokines, most effectively increased the expression level of the *iNOS* gene. A high expression level of the *IDO* gene was observed in the group of hUCB-MSCs preconditioned with the combination of cytokines IL-17+TNF-α. At the same time, a significant increase in *COX-2* gene expression was observed in the hUCB-MSC group preconditioned with cytokine IL-22 alone. Preconditioning of hUCB-MSCs with the combination of cytokines IL-17+TNF-α+IL-22 most effectively increased the level of expression of the *HGF* gene. A significant increase in the expression level of the *TSG-6* gene was observed only in the group of hUCB-MSCs preconditioned with the cytokine IL-17. An increase in the expression level of the *IL-10* was observed in the group of hUCB-MSCs preconditioned with the cytokine IL-22 alone, but the most effective increase was noted in the group treated with the combination of cytokines IL-17+TNF-α. Preconditioning of hUCB-MSCs with the combination of cytokines TNF-α+IL-22 leads to a significant increase in the expression level of *TGFβ*. Finally, preconditioning of hUCB-MSCs with the cytokine combination IL-17+TNF-α+IL-22 effectively increased the expression level of the *Gal-1* gene. According to the literature, it was found that preconditioning with a combination of cytokines IL-22, TNF-a, and IFN-γ enhanced the proliferation and migration of MSCs. Preconditioning with IL-22 increased genes responsible for osteogenic and adipogenic differentiation [100]. Furthermore, preconditioning of UCB-MSC with TNF-α revealed significant expression of *HGF*, *IDO*, *TGF-β1*, and *COX-2* genes, but the level of *COX-1* was moderate, while *IL-10* was not detected. In addition, authors observed that preconditioning of UCB-MSC with IFN-γ along or in combination with TNF-α for more than 72 h still resulted in noteworthy expression of the *IDO* gene [101]. The levels of markers associated with inflammation, namely *IL-8*, *IL-6*, *CXCL10*, and *COX-2*, were decreased in a psoriasis-like 3D reconstructed skin model by the effect of exosomes from mononuclear cells of human umbilical cord blood (UCB-MNC-sEV). UCB-MNC-sEV was applied in the quantity of 1 × 10^10^ particles/ml two times daily, for a total 6 days. In this case, MSCs themselves were not used as a source of the exosomes; however, the effect of exosomes from mononuclear cells of human UCB was remarkable [55]. Another study by Chen et al. assessed mRNA levels of inflammatory cytokines and keratinocyte differentiation markers in a mouse model of imiquimod (IMQ)-induced psoriasis. Total RNA was extracted from mouse dorsal skin, isolated plasmacytoid dendritic cells (pDCs), and splenic neutrophils. Gene expression analysis revealed significant upregulation of proinflammatory cytokines *IL-17*, *IL-23*, *IL-6*, and *IL-1β* in the skin of psoriatic mice. Conversely, the anti-inflammatory cytokine IL-10 was significantly downregulated. Upon MSC infusion, there was a substantial decrease in the mRNA levels of proinflammatory cytokines and keratinocyte markers. Specifically, the expression levels of *IL-17*, *IL-23*, *IL-6*, and *IL-1β* were significantly reduced compared to those in untreated psoriatic mice. Importantly, *IL-10* expression was significantly upregulated following MSC treatment. These results suggest that MSCs may modulate the inflammatory environment by downregulating inflammation-related genes and upregulating genes associated with anti-inflammatory responses [102]. It was reported that infusion of adoptively transferred murine MSCs derived from bone marrow and adipose tissue resulted in significant upregulation of *IL-17A* and transforming growth factor-beta (*TGF-β*) mRNA levels in the skin compared to PBS-treated controls in the model of IMQ-treated mice. Although *IL-17A* is a proinflammatory cytokine, its increased expression in this context may reflect a prominent role in tissue repair and immune regulation mediated by MSCs [103]. Interestingly, IL-17-preconditioned MSCs express genes involved in the chemotaxis process. Enhanced levels of matrix metalloproteinases (*MMP1*, *MMP13*, and *CXCL6*) were also detected in MSCs pretreated with IL-17 [104]. It was reported that preconditioning with IL-17 activated the relocation of MSCs and cells that were involved in inflammation, as well as the recognition of lymphocytes with produced anti-inflammatory molecules of MSCs. The level of gene expression of immunosuppressive *COX-2* was higher than in controls in MSCs that were co-cultured with a one-way mixed leukocyte reaction (MLR) (activated T cells) [105]. It was also reported that hUCB-MSCs reduce the expression levels of proinflammatory cytokine and chemokine genes, including *CXCL1*, *CCL17*, and *CCL20* [106].

Other mechanisms contributing to the pathogenesis of psoriasis include inflammation-associated keratinocyte death (necroptosis), altered STAT1 signaling, and upregulation of several chemokine genes such as C-C chemokine receptor type 7 (*CCR7*), C-C motif ligand 2 (*CCL2*), *CCL19*, CXC motif chemokine ligand 8 (*CXCL8*), *CXCL1*, and *CXCL2* [107,108,109]. Necroptosis, a form of regulated necrosis mediated by receptor-interacting protein kinase 1 (RIPK1), RIPK3, and mixed-lineage kinase domain-like pseudokinase (MLKL), plays a central role in the inflammatory cascade of psoriasis and is also implicated in other inflammatory diseases [107]. STAT1 is another important regulator: its downregulation promotes STAT3 activation and IL-22 production, whereas activation of STAT1 suppresses skin inflammation in imiquimod-induced psoriasis models [108]. Chemokines encoded by *CCR7*, *CCL2*, *CCL19*, *CXCL8*, *CXCL1*, and *CXCL2* genes have been identified as potential biomarkers of psoriasis [109]. Among them, *CXCL1* is particularly relevant, as it is upregulated in psoriatic lesions and serum, where it promotes neutrophil migration and amplifies inflammation [110]. In contrast, the role of charged multivesicular body protein 2B (CHMP2B) in psoriasis remains unconfirmed. Therapeutically, MSC-EVs can attenuate necroptosis by delivering specificity protein 1 (SP1) and other bioactive molecules that activate sphingosine kinase 1–sphingosine-1-phosphate (SK1–S1P) signaling in recipient cells, thereby reducing RIPK3 and MLKL phosphorylation [111]. In addition, MSCs suppress Th17 cell differentiation through IFN-γ-mediated STAT1 activation, which upregulates *SOCS3* and inhibits STAT3 signaling, ultimately exerting immunomodulatory effects in autoimmune diseases, including psoriasis [112]. Furthermore, it has been reported that neutrophil infiltration, a hall-mark of psoriasis, is markedly reduced following treatment with IFN-γ/TNF-α-preconditioned MSCs (MSCs-IT). This effect is mediated by *TSG-6*, whose expression is induced by these cytokines and which suppresses neutrophil recruitment by downregulating *CXCL1*, likely via reduced STAT1 phosphorylation in keratinocytes [113].

One of the main objectives of our work was to compare the therapeutic efficacy of subcutaneous injections of both intact and preconditioned hUCB-MSCs and their exosomes (hUCB-MSC-Exo) in a model of IMQ-induced psoriasis-like inflammation in mice. For this purpose, after preconditioning, we isolated hUCB-MSC-Exo from both intact and preconditioned hUCB-MSCs using a differential ultracentrifugation technique. Ultracentrifugation, the gold standard for exosome isolation, allows for the production of highly enriched exosome fractions [114]. In this study, the size of isolated hUCB-MSC-Exo was within the characteristic exosomal range (30–200 nm), with an average diameter of 85.34 nm according to the NanoBrook 90Plus Zeta analyzer and 95.93 nm based on SEM analysis [32,115,116,117,118,119,120,121,122]. Moreover, the zeta potential measurements showed that the surface charge of hUCB-MSC-Exo was between −21.24 mV and −25.16 mV, which is consistent with data reported in the literature. For instance, Li et al. measured the zeta potential of MSC-derived EVs (including exosomes, microvesicles, and apoptotic bodies) and reported a value of approximately −26.3 mV [123]. Similarly, de Almeida Fuzeta et al. reported that the surface charge of EVs, including exosomes and microvesicles, was −15.5 ± 1.6 mV and −19.4 ± 1.4 mV, respectively [124]. In another study, Zhang et al. reported an average zeta potential of −42.06 mV for MSC-Exo [125]. Furthermore, the isolated hUCB-MSC-Exo samples exhibited expression of the exosomal marker CD9, as confirmed by Western blot analysis, further validating the success of exosome isolation. It is well known that the most prevalent exosome surface markers are CD9, CD63, and CD81, which are members of the tetraspanin protein family [126,127,128]. At the same time, hUCB-MSC-Exo express exosome-specific transmembrane protein markers such as CD9, CD63, CD81, tumor susceptibility gene 101 (TSG101), and apoptosis-linked-gene-2-interacting protein X (ALIX) and do not express calnexin and cytochrome C [122,129].

Psoriasis is a common immune-mediated chronic inflammatory skin disease that causes erythematous, itchy, scaly patches and is characterized by a high incidence, long duration, and a tendency to relapse [130]. Psoriasis is caused by an imbalance of Th1/Th17 chemokines and cytokines such as IL-17, IL-23, TNFα, and IFN-γ, which can be explained as dysfunction of keratinocytes, immune cells, and inflammatory cells [4]. Psoriasis was initially associated with Th1 cells and their cytokines, including TNF-α and IFN-γ [131]. Many studies have shown that Th17 cells and their inflammatory mediators play a significant role in the pathophysiology of psoriasis [132]. Th17 cytokines, such as IL-6, IL-17A, IL-17F, IL-21, and IL22, stimulate keratinocyte activation and overproliferation [133,134]. Biopsy specimens from plaques of psoriasis vulgaris demonstrate increased levels of IL-17 in association with increased expression of IL-23 and IL-22, whereas serum levels of IL-17 are associated with psoriasis severity [135,136,137,138]. IL-22 is another important downstream cytokine in the IL-23/Th17 axis that is overexpressed in psoriatic skin compared to normal skin [139,140,141,142]. IL-22 causes keratinocyte hyperplasia by activating signal transducer and activator of transcription 3 (STAT3), which leads to psoriasiform hyperplasia. In the absence of IL-22, the severity of both IL-23-mediated and IMQ-induced psoriasis-like dermatitis in relevant mice models is significantly reduced [61,141,143]. As a consequence, activation of keratinocytes promotes the recruitment of inflammatory cells [133,135]. Today, MSC-based therapy is used to treat several pathological conditions, including bone and cartilage diseases, cardiac ischemia, diabetes, and neurological disorders. Along with MSCs, the study of the therapeutic properties of MSC-Exo is promising. It is not surprising that there is a high interest in studying the therapeutic properties of MSCs and MSC-Exo in the treatment of psoriasis [144]. Several studies have demonstrated that MSCs and MSC-Exo can effectively treat psoriasis-like skin lesions in mouse models [145]. For instance, subcutaneous injection of hUCB-MSCs ameliorated IMQ-induced and IL-23-mediated psoriasis-like skin inflammation [106]. It has been shown that human umbilical cord MSC-derived exosomes (hUCMSCs-Exo) are able to alleviate psoriasis-like skin inflammation by suppressing the expression of IL-17, IL-23, CCL20, and STAT3 [53]. Administration of human umbilical cord blood mononuclear cell-derived exosomes (UCB-MNC-Exo) increases the number of Tregs in skin and prevents acanthosis in an IMQ-induced psoriasis-like inflammation model [55]. This is the first study to compare the therapeutic potential of intact and cytokine-preconditioned (IL-17, IL-22, and TNF-α) hUCB-MSCs and hUCB-MSC-Exo in a mouse model of IMQ-induced psoriasis-like skin inflammation. Our results showed that significant attenuation of psoriasis symptoms including reduction in erythema, scaling, and skin thickness was observed in the following groups: IMQ + MSC, IMQ + MSC-2C preconditioned with IL-22+TNF-α cytokines, and IMQ + MSC-Exo 3C preconditioned with IL-17, IL-22, and TNF-α cytokines. The best therapeutic effect was observed in the IMQ + MSC-Exo 3C group, where the cumulative PASI score was 4—65.2% lower than in untreated mice (11.5). Thus, intact hUCB-MSCs, MSC-2C, and MSC-Exo 3C exert a protective effect against IMQ-induced psoriasis-like skin inflammation.

The spleen is an important organ of the immune system that secretes a number of immune-active cytokines, so it plays an important role in the functioning of the immune system [145]. In this study, we observed that mice treated with IMQ alone had significantly increased spleen size, which is a clear sign of splenomegaly. Splenomegaly is a typical phenomenon of inflammation [146]. It has been reported that long-term psoriasis patients also experience an increase in spleen diameter, which is a consequence of the immune system’s response to a state of chronic inflammation [147]. We found that MSC-3C and MSC-Exo 3C slightly inhibited the spleen-to-body weight ratio, indicating their ability to regulate spleen inflammatory immune cells and have a systemic antipsoriatic effect. Furthermore, we measured the percentages of CTLs, Th cells, and Tregs in the mouse spleen, since there is evidence that MSCs are able to suppress the secretion of cytokines, the cytotoxic activity of proinflammatory CTLs, and the proliferation and proinflammatory properties of Th cells and stimulate Treg proliferation and their inhibitory abilities [148]. It is well known that in psoriasis, both skin and peripheral blood are infiltrated by CTLs and Th cells, which produce elevated levels of proinflammatory cytokines [149]. CTLs secrete IL-2, IFN-γ, TNF-α, IL-17, and IL-22, which contribute to psoriasis development. Early in the psoriatic cascade, IFN-γ activates antigen-presenting cells (APCs) and keratinocytes, inducing IL-22 and IL-1β production and amplifying inflammation. TNF-α regulates APCs, stimulates dendritic cells to release IL-23, and synergizes with cytokines such as IL-17A to enhance the inflammatory cascade and promote T cell proliferation and migration to lesions. Th cells are divided into Th1, Th2, Th17, and Th22 cells, each of which has different functions. Th1 cells enhance immune responses by releasing proinflammatory cytokines such as IFN-γ and TNF-α, which are crucial in psoriasis development. Th2 cells produce IL-4 and IL-10, Th17 cells secrete IL-17, TNF-α, IL-6, IL-21, and IL-22, while Th22 cells release IL-12 and IL-13. These cytokines synergistically stimulate the chronic inflammatory environment underlying the pathogenesis of psoriasis [150]. The pathogenesis of psoriasis is largely due to cytokine-producing Th1 and Th17 cells, which are normally controlled by Tregs. The suppressive function of Tregs is mediated through cell–cell interactions, the production of inhibitory cytokines (IL-10, TGF-β1, and IL-35), and direct cytotoxic activity. In psoriasis, impaired Treg function leads to an imbalance in the Th17/Treg ratio [151]. This dysfunction is driven by IL-6, IL-21, and IL-23, which induce STAT3 phosphorylation, highlighting the role of the proinflammatory cytokine environment in impairing Treg activity [150]. In our study the percentage of CTLs decreased in the IMQ + MSC 2C group, while in all other groups, it increased. A decrease in the Th cell ratio was observed in almost all groups receiving cell or exosome therapy, with the greatest reduction in the IMQ + MSC-Exo 3C group. Meanwhile, the percentage of Tregs increased significantly in the intact IMQ + MSC and IMQ + MSC-Exo 3C treatment groups. Our results suggest that intact and preconditioned hUCB-MSCs and their exosomes have immunomodulatory and anti-inflammatory properties. Finally, histopathological examination (H&E staining) confirmed that intact hUCB-MSCs, MSC-3C, and MSC-Exo 3C could prevent the proliferation and abnormal differentiation of keratinocytes. According to the Baker scoring system, MSC-Exo 3C treatment showed the best results, highlighting its potential as a therapeutic approach for psoriasis-like skin lesions.

Limitations of our study include the fact that the results are based only on experiments conducted in a mouse model of psoriasis, without validation using independent datasets or clinically obtained patient samples. Although this model reliably recapitulates key aspects of psoriatic inflammation, the lack of clinical validation limits the direct translation of our results to human disease. Further studies involving independent cohorts and clinical samples will be critical to confirm the therapeutic potential of preconditioned hUCB-MSCs and hUCB-MSC-Exo in the treatment of psoriasis.

## 5. Conclusions

The data suggest that subcutaneous administration of exosomes derived from hUCB-MSCs preconditioned with IL-17, IL-22, and TNF-α has therapeutic potential for treating skin inflammation and could be applied in psoriasis treatment.

## Figures and Tables

**Table 1:** RT-PCR primer sequence.

No.	Gene Name	Primers
1	Cy5-*ACTB*-Pr-BH Q3	ACTCTTCCAGCCTTCCTTCC
		Forward: TCACCATTGGCAATGAG
		Revers: CCACGTCACACTTCATG
2	FAM-*HGF*-BHQ1	TCACGAGCATGACATGACTC
		Forward: GTTGGGATTCTCAGTATC
		Revers: CACGATAACAATCTTGTC
3	FAM-*TGF-β*-BHQ1	CGCACGCAGCAGTTCTTCTC
		Forward: ACACCAACTATTGCTTCA
		Revers: CTTGCGGAAGTCAATGTA
4	FAM-*IDO*-BHQ1	TTCCTTACTGCCAACTCTCCAAGAA
		Forward: CTTGCCAAGAAATATTGC
		Revers: CGTCCATGTTCTCATAAG
5	FAM-*iNOS*-BHQ1	CAGCAAGCAGCAGAATGAGTCC
		Forward: GACCTTCAGTATCACAAC
		Revers: GTGTCTTGGAAAGTCATC
6	FAM-*Galectin-1*-BHQ1	TCTTAGCGTCAGGAGCCACC
		Forward: CCTGAATCTCAAACCTGGA
		Revers: GGTTGTTGCTGTCTTTGC
7	FAM-*IL-10*-BHQ1	CTCAGACAAGGCTTGGCAACC
		Forward: AGCAGAGTGAAGACTTTC
		Revers: CTCCTCCAGGTAAAACTG
8	FAM-*COX2*-BHQ1	ACTATCTGCTTCATCCGCCAACTAA
		Forward: CGTCATTATTGGCTCAAC
		Revers: GATGGAGACATACAGAAATAG

**Table 2:** Histopathological scoring of severity of inflammation (Baker scoring system).

Layers	Feature	Score
Keratin	Munro abscess	2.0
	Hyperkeratosis	0.5
	Parakeratosis	1.0
Epidermis	Thinning above papillae	0.5
	Rete ridges appearance	1.5
	Acanthosis	0.5
	Lack of granular layer	1.0
Dermis	Lymphocytic infiltrate	
	Mild	0.5
	Moderate	1.0
	Severe	2.0
	Papillary congestion	0.5

## Data Availability

The data presented in this study are available on request from the corresponding author.

## Supplementary Materials

The following supporting information can be downloaded at: https://www.mdpi.com/article/10.3390/biology14081033/s1, Figure S1. Western blot analysis confirming the presence of the CD9 protein on the surface of hUCB-MSC-Exo.

## References

[1] Menter A., Gottlieb A., Feldman S.R., Van Voorhees A.S., Leonardi C.L., Gordon K.B., Lebwohl M., Koo J.Y.M., Elmets C.A., Korman N.J. (2008). Guidelines of care for the management of psoriasis and psoriatic arthritis: Section 1. Overview of psoriasis and guidelines of care for the treatment of psoriasis with biologics. J. Am. Acad. Dermatol..

[2] Sewerin P., Brinks R., Schneider M., Haase I., Vordenbäumen S. (2019). Prevalence and incidence of psoriasis and psoriatic arthritis. Ann. Rheum. Dis..

[3] Dairov A., Issabekova A., Sekenova A., Shakhatbayev M., Ogay V. (2024). Prevalence, incidence, gender and age distribution, and economic burden of psoriasis worldwide and in Kazakhstan. J. Clin. Med. Kaz..

[4] Griffiths C.E.M., Armstrong A.W., Gudjonsson J.E., Barker J.N.W.N. (2021). Psoriasis. Lancet.

[5] Singh A., Easwari T.S. (2021). Recent advances in psoriasis therapy: Trends and future prospects. Curr. Drug Targets.

[6] Nestle F.O., Kaplan D.H., Barker J. (2009). Psoriasis. N. Engl. J. Med..

[7] Hawkes J.E., Yan B.Y., Chan T.C., Krueger J.G. (2018). Discovery of the IL-23/IL-17 signaling pathway and the treatment of psoriasis. J. Immunol..

[8] Lee H.-J., Kim M. (2023). Challenges and future trends in the treatment of psoriasis. Int. J. Mol. Sci..

[9] Raharja A., Mahil S.K., Barker J.N. (2021). Psoriasis: A brief overview. Clin. Med..

[10] Zhang B., Lai R.C., Sim W.K., Choo A.B.H., Lane E.B., Lim S.K. (2021). Topical application of mesenchymal stem cell exosomes alleviates the imiquimod induced psoriasis-like inflammation. Int. J. Mol. Sci..

[11] Vasanthan J., Gurusamy N., Rajasingh S., Sigamani V., Kirankumar S., Thomas E.l., Rajasingh J. (2020). Role of human mesenchymal stem cells in regenerative therapy. Cells.

[12] Alvites R., Branquinho M., Sousa A.C., Lopes B., Sousa P., Maurício A.C. (2022). Mesenchymal stem/stromal cells and their paracrine activity—Immunomodulation mechanisms and how to influence the therapeutic potential. Pharmaceutics.

[13] García-Bernal D., García-Arranz M., Yáñez R.M., Hervás-Salcedo R., Cortés A., Fernández-García M., Hernando-Rodríguez M., Quintana-Bustamante Ó., Bueren J.A., García-Olmo D. (2021). The current status of mesenchymal stromal cells: Controversies, unresolved issues and some promising solutions to improve their therapeutic efficacy. Front. Cell Dev. Biol..

[14] Saeedi P., Halabian R., Fooladi A.A.I. (2019). A revealing review of mesenchymal stem cells therapy, clinical perspectives and Modification strategies. Stem Cell Investig..

[15] Aravindhan S., Ejam S.S., Lafta M.H., Markov A., Yumashev A.V., Ahmadi M. (2021). Mesenchymal stem cells and cancer therapy: Insights into targeting the tumour vasculature. Cancer Cell Int..

[16] Ogay V., Sekenova A., Li Y., Issabekova A., Saparov A. (2021). The therapeutic potential of mesenchymal stem cells in the treatment of atherosclerosis. Curr. Stem Cell Res. Ther..

[17] Bakinowska E., Bratborska A.W., Kiełbowski K., Ćmil M., Biniek W.J., Pawlik A. (2024). The role of mesenchymal stromal cells in the treatment of rheumatoid arthritis. Cells.

[18] Sarsenova M., Issabekova A., Abisheva S., Rutskaya-Moroshan K., Ogay V., Saparov A. (2021). Mesenchymal stem cell-based therapy for rheumatoid arthritis. Int. J. Mol. Sci..

[19] Zhang Y., Fan M., Zhang Y. (2024). Revolutionizing bone defect healing: The power of mesenchymal stem cells as seeds. Front. Bioeng. Biotechnol..

[20] Wu S., Sun S., Fu W., Yang Z., Yao H., Zhang Z. (2024). The role and prospects of mesenchymal stem cells in skin repair and regeneration. Biomedicines.

[21] Maxson S., Lopez E.A., Yoo D., Danilkovitch-Miagkova A., Leroux M.A. (2012). Concise review: Role of mesenchymal stem cells in wound repair. Stem Cells Transl. Med..

[22] Pittenger M.F., Discher D.E., Péault B.M., Phinney D.G., Hare J.M., Caplan A.I. (2019). Mesenchymal stem cell perspective: Cell biology to clinical progress. NPJ Regen. Med..

[23] Merimi M., El-Majzoub R., Lagneaux L., Moussa Agha D., Bouhtit F., Meuleman N., Fahmi H., Lewalle P., Fayyad-Kazan M., Najar M. (2021). The therapeutic potential of mesenchymal stromal cells for regenerative medicine: Current knowledge and future understandings. Front. Cell Dev. Biol..

[24] Fu Y., Karbaat L., Wu L., Leijten J., Both S.K., Karperie M. (2017). Trophic effects of mesenchymal stem cells in tissue regeneration. Tissue Eng. Part B Rev..

[25] Rhee K.-J., Lee J.I., Eom Y.W. (2015). Mesenchymal stem cell-mediated effects of tumor support or suppression. Int. J. Mol. Sci..

[26] Ayala-Cuellar A.P., Kang J.-H., Jeung E.-B., Choi K.-C. (2019). Roles of mesenchymal stem cells in tissue regeneration and immunomodulation. Biomol. Ther..

[27] Musiał-Wysocka A., Kot M., Majka M. (2019). The pros and cons of mesenchymal stem cell-based therapies. Cell Transplant..

[28] Zhou T., Yuan Z., Weng J., Pei D., Du X., He C., Lai P. (2021). Challenges and advances in clinical applications of mesenchymal stromal cells. J. Hematol. Oncol..

[29] Volarevic V., Markovic B.S., Gazdic M., Volarevic A., Jovicic N., Arsenijevic N., Armstrong L., Djonov V., Lako M., Stojkovic M. (2018). Ethical and safety issues of stem cell-based therapy. Int. J. Med. Sci..

[30] Mastrolia I., Foppiani E.M., Murgia A., Candini O., Samarelli A.V., Grisendi G., Veronesi E., Horwitz E.M., Dominici M. (2019). Challenges in clinical development of mesenchymal stromal/stem cells: Concise review. Stem Cells Transl. Med..

[31] Ancans J. (2012). Cell therapy medicinal product regulatory framework in Europe and its application for MSC-based therapy development. Front. Immunol..

[32] Riazifar M., Pone E.J., Lötvall J., Zhao W. (2017). Stem cell extracellular vesicles: Extended messages of regeneration. Annu. Rev. Pharmacol. Toxicol..

[33] Li X., Corbett A.L., Taatizadeh E., Tasnim N., Little J.P., Garnis C., Daugaard M., Guns E., Hoorfar M., Li I.T.S. (2019). Challenges and opportunities in exosome research-Perspectives from biology, engineering, and cancer therapy. APL Bioeng..

[34] Samavati S.F., Yarani R., Kiani S., HoseinKhani Z., Mehrabi M., Levitte S., Primavera R., Chetty S., Thakor A.S., Mansouri K. (2024). Therapeutic potential of exosomes derived from mesenchymal stem cells for treatment of systemic lupus erythematosus. J. Inflamm..

[35] Tan F., Li X., Wang Z., Li J., Shahzad K., Zheng J. (2024). Clinical applications of stem cell-derived exosomes. Signal Transduct. Target. Ther..

[36] Clua-Ferré L., Suau R., Vañó-Segarra I., Ginés I., Serena C., Manyé J. (2024). Therapeutic potential of mesenchymal stem cell-derived extracellular vesicles: A focus on inflammatory bowel disease. Clin. Transl. Med..

[37] Kordelas L., Rebmann V., Ludwig A.-K., Radtke S., Ruesing J., Doeppner T.R., Epple M., Horn P.A., Beelen D.W., Giebel B. (2014). MSC-derived exosomes: A novel tool to treat therapy-refractory graft-versus-host disease. Leukemia.

[38] Salehpour A., Karimi Z., Zadeh M.G., Afshar M., Kameli A., Mooseli F., Zare M., Afshar A. (2024). Therapeutic potential of mesenchymal stem cell-derived exosomes and miRNAs in neuronal regeneration and rejuvenation in neurological disorders: A mini review. Front. Cell. Neurosci..

[39] Kråkenes T., Sandvik C.E., Ytterdal M., Gavasso S., Evjenth E.C., Bø L., Kvistad C.E. (2024). The therapeutic potential of exosomes from mesenchymal stem cells in multiple sclerosis. Int. J. Mol. Sci..

[40] Akhlaghpasand M., Tavanaei R., Hosseinpoor M., Yazdani K.O., Soleimani A., Zoshk M.Y., Soleimani M., Chamanara M., Ghorbani M., Deylami M. (2024). Safety and potential effects of intrathecal injection of allogeneic human umbilical cord mesenchymal stem cell-derived exosomes in complete subacute spinal cord injury: A first-in-human, single-arm, open-label, phase I clinical trial. Stem Cell Res. Ther..

[41] Civelek E., Kabatas S., Savrunlu E.C., Diren F., Kaplan N., Ofluoğlu D., Karaöz E. (2024). Effects of exosomes from mesenchymal stem cells on functional recovery of a patient with total radial nerve injury: A pilot study. World J. Stem Cells.

[42] Jiang M., Jiang X., Li H., Zhang C., Zhang Z., Wu C., Zhang J., Hu J., Zhang J. (2023). The role of mesenchymal stem cell-derived EVs in diabetic wound healing. Front. Immunol..

[43] Lu X., Guo H., Wei W., Lu D., Shu W., Song Y., Qiu N., Xu X. (2023). Current status and prospect of delivery vehicle based on mesenchymal stem cell-derived exosomes in liver diseases. Int. J. Nanomed..

[44] Zamanian M.H., Norooznezhad A.H., Hosseinkhani Z., Hassaninia D., Mansouri F., Vaziri S., Payandeh M., Heydarpour F., Kiani S., Shirvani M. (2024). Human placental mesenchymal stromal cell-derived small extracellular vesicles as a treatment for severe COVID-19: A double-blind randomized controlled clinical trial. J. Extracell. Vesicles.

[45] Chu M., Wang H., Bian L., Huang J., Wu D., Zhang R., Fei F., Chen Y., Xia J. (2022). Nebulization therapy with umbilical cord mesenchymal stem cell-derived exosomes for COVID-19 pneumonia. Stem Cell Rev. Rep..

[46] Zhu Y.-G., Shi M.-M., Monsel A., Dai C.-X., Dong X., Shen H., Li S.-K., Chang J., Xu C.-L., Li P. (2022). Nebulized exosomes derived from allogenic adipose tissue mesenchymal stromal cells in patients with severe COVID-19: A pilot study. Stem Cell Res. Ther..

[47] Sengupta V., Sengupta S., Lazo A., Woods P., Nolan A., Bremer N. (2020). Exosomes derived from bone marrow mesenchymal stem cells as treatment for severe COVID-19. Stem Cells Dev..

[48] Shamili F.H., Bayegi H.R., Salmasi Z., Sadrim K., Mahmoudi M., Kalantari M., Ramezani M., Abnous K. (2018). Exosomes derived from TRAIL-engineered mesenchymal stem cells with effective anti-tumor activity in a mouse melanoma model. Int. J. Pharm..

[49] de Araujo Farias V., O’Valle F., Serrano-Saenz S., Anderson P., Andrés E., López-Peñalver J., Tovar I., Nieto A., Santos A., Martín F. (2018). Exosomes derived from mesenchymal stem cells enhance radiotherapy-induced cell death in tumor and metastatic tumor foci. Mol. Cancer.

[50] Cho B.S., Kim J.O., Ha D.H., Yi Y.W. (2018). Exosomes derived from human adipose tissue-derived mesenchymal stem cells alleviate atopic dermatitis. Stem Cell Res. Ther..

[51] Shin K.-O., Ha D.H., Kim J.O., Crumrine D.A., Meyer J.M., Wakefield J.S., Lee Y., Kim B., Kim S., Kim H.-K. (2020). Exosomes from human adipose tissue-derived mesenchymal stem cells promote epidermal barrier repair by inducing *de novo* synthesis of ceramides in atopic dermatitis. Cells.

[52] Zhang M., Johnson-Stephenson T.K., Wang W., Wang Y., Li J., Li L., Zen K., Chen X., Zhu D. (2022). Mesenchymal stem cell-derived exosome-educated macrophages alleviate systemic lupus erythematosus by promoting efferocytosis and recruitment of IL-17+ regulatory T cell. Stem Cell Res. Ther..

[53] Zhang Y., Yan J., Li Z., Zheng J., Sun Q. (2022). Exosomes derived from human umbilical cord mesenchymal stem cells alleviate psoriasis-like skin inflammation. J. Interf. Cytokine Res..

[54] Xu F., Fei Z., Dai H., Xu J., Fan Q., Shen S., Zhang Y., Ma Q., Chu J., Peng F. (2022). Mesenchymal stem cell-derived extracellular vesicles with high PD-L1 expression for autoimmune diseases treatment. Adv. Mater..

[55] Rodrigues S.C., Cardoso R.M.S., Freire P.C., Gomes C.F., Duarte F.V., das Neves R.P., Simões-Correia J. (2021). Immunomodulatory properties of umbilical cord blood-derived small extracellular vesicles and their therapeutic potential for inflammatory skin disorders. Int. J. Mol. Sci..

[56] Kim H.-S., Shin T.-H., Lee B.-C., Yu K.-R., Seo Y., Lee S., Seo M., Hong I., Choi S.W., Seo K. (2013). Human umbilical cord blood mesenchymal stem cells reduce colitis in mice by activating NOD2 signaling to COX2. Gastroenterology.

[57] Reger R.L., Tucker A.H., Wolfe M.R., Prockop D.J., Bunnell B.A. (2008). Differentiation and characterization of human MSCs. Mesenchymal Stem Cells Methods in Molecular Biology.

[58] Baliwag J., Barnes D.H., Johnston A. (2015). Cytokines in psoriasis. Cytokine.

[59] Guo J., Zhang H., Lin W., Lu L., Su J., Chen X. (2023). Signaling pathways and targeted therapies for psoriasis. Signal Transduct. Target. Ther..

[60] Gupta S., Rawat S., Arora V., Kottarath S.K., Dinda A.K., Vaishnav P.K., Nayak B., Mohanty S. (2018). An improvised one-step sucrose cushion ultracentrifugation method for exosome isolation from culture supernatants of mesenchymal stem cells. Stem Cell Res. Ther..

[61] van der Fits L., Mourits S., Voerman J.S.A., Kant M., Boon L., Laman J.D., Cornelissen F., Mus A.-M., Florencia E., Prens E.P. (2009). Imiquimod-induced psoriasis-like skin inflammation in mice is mediated via the IL-23/IL-17 axis. J. Immunol..

[62] Salwa F., Badanthadka M., D’Souza L. (2021). Differential psoriatic effect of imiquimod on Balb/c and Swiss mice. J. Health Allied SciNU..

[63] Gray E.E., Ramírez-Valle F., Xu Y., Wu S., Wu Z., Karjalainen K.E., Cyster J.G. (2013). Deficiency in IL-17-committed Vγ4(+) γδ T cells in a spontaneous Sox13-mutant CD45.1(+) congenic mouse substrain provides protection from dermatitis. Nat. Immunol..

[64] Moos S., Mohebiany A.N., Waisman A., Kurschus F.C. (2019). Imiquimod-induced psoriasis in mice depends on the IL-17 signaling of keratinocytes. J. Investig. Dermatol..

[65] Grosjean C., Quessada J., Nozais M., Loosveld M., Payet-Bornet D., Mionnet C. (2021). Isolation and enrichment of mouse splenic T cells for ex vivo and in vivo T cell receptor stimulation assays. STAR Protoc..

[66] Baker B.S., Brent L., Valdimarsson H., Powles A.V., al-Imara L., Walker M., Fry L. (1992). Is epidermal cell proliferation in psoriatic skin grafts on nude mice driven by T-cell derived cytokines?. Br. J. Dermatol..

[67] Mohammed S.S., Kadhim H.M., Al-Sudani I.M., Mustafa W.W. (2022). Study the topical effect of six days use of different lycopene doses on imiquimod-induce psoriasis-like skin inflammation in mice. Int. J. Health Sci..

[68] De Rosa G., Mignogna C. (2007). The histopathology of psoriasis. Reumatismo.

[69] Camilleri E.T., Gustafson M.P., Dudakovic A., Riester S.M., Garces C.G., Paradise C.R., Takai H., Karperien M., Cool S., Sampen H.-J.I. (2016). Identification and validation of multiple cell surface markers of clinical-grade adipose-derived mesenchymal stromal cells as novel release criteria for good manufacturing practice-compliant production. Stem Cell Res. Ther..

[70] Sabat R., Philipp S., Höflich C., Kreutzer S., Wallace E., Asadullah K., Volk H., Sterry W., Wolk K. (2007). Immunopathogenesis of psoriasis. Exp. Dermatol..

[71] Lawrence D.A. (1991). Identification and activation of latent transforming growth factor beta. Methods Enzymol..

[72] Han S.-W., Kim T.-Y., Hwang P.G., Jeong S., Kim J., Choi I.S., Oh D.-Y., Kim J.H., Kim D.-W., Chung D.H. (2005). Predictive and prognostic impact of epidermal growth factor receptor mutation in non-small-cell lung cancer patients treated with gefitinib. J. Clin. Oncol..

[73] Flisiak I., Chodynicka B., Porebski P., Flisiak R. (2002). Association between psoriasis severity and transforming growth factor beta(1) and beta (2) in plasma and scales from psoriatic lesions. Cytokine.

[74] Flisiak I., Zaniewski P., Chodynicka B. (2008). Plasma TGF-beta1, TIMP-1, MMP-1 and IL-18 as a combined biomarker of psoriasis activity. Biomarkers.

[75] Meki A.-R.M.A., Al-Shobaili H. (2014). Serum vascular endothelial growth factor, transforming growth factor β1, and nitric oxide levels in patients with psoriasis vulgaris: Their correlation to disease severity. J. Clin. Lab. Anal..

[76] de Araújo Farias V., Carrillo-Gálvez A.B., Martín F., Anderson P. (2018). TGF-β and mesenchymal stromal cells in regenerative medicine, autoimmunity and cancer. Cytokine Growth Factor Rev..

[77] He Q., Shi J., Liu W., Zhao W., Wang Z., Liu K., Zhao D., Wang S., Guo Y., Cheng L. (2022). TGF-β1-induced bone marrow mesenchymal stem cells (BMSCs) migration via histone demethylase KDM6B mediated inhibition of methylation marker H3K27me3. Cell Death Discov..

[78] Liu F., Qiu H., Xue M., Zhang S., Zhang X., Xu J., Chen J., Yang Y., Xie J. (2019). MSC-secreted TGF-β regulates lipopolysaccharide-stimulated macrophage M2-like polarization via the Akt/FoxO1 pathway. Stem Cell Res. Ther..

[79] Bonifati C., Ameglio F. (1999). Cytokines in psoriasis. Int. J. Dermatol..

[80] Cutroneo K.R. (2007). TGF-beta-induced fibrosis and SMAD signaling: Oligo decoys as natural therapeutics for inhibition of tissue fibrosis and scarring. Wound Repair Regen..

[81] Han G., Williams C.A., Salter K., Garl P.J., Li A.G., Wang X.-J. (2010). A role for TGF beta signaling in the pathogenesis of psoriasis. J. Investig. Dermatol..

[82] Kerkis I., da Silva Á.P., Araldi R.P. (2024). The impact of interleukin-6 (IL-6) and mesenchymal stem cell-derived IL-6 on neurological conditions. Front. Immunol..

[83] Saggini A., Chimenti S., Chiricozzi A. (2014). IL-6 as a druggable target in psoriasis: Focus on pustular variants. J. Immunol. Res..

[84] Blauvelt A. (2017). IL-6 differs from TNF-α: Unpredicted clinical effects caused by IL-6 blockade in psoriasis. J. Investig. Dermatol..

[85] Neurath M.F., Finotto S. (2011). IL-6 signaling in autoimmunity, chronic inflammation and inflammation-associated cancer. Cytokine Growth Factor Rev..

[86] Pietrzak A., Chabros P., Grywalska E., Pietrzak D., Kandzierski G., Wawrzycki B., Roliński J., Gawęda K., Krasowska D. (2020). Serum concentration of interleukin 6 is related to inflammation and dyslipidemia in patients with psoriasis. Postepy Dermatol. Alergol..

[87] Pricola K.L., Kuhn N.Z., Haleem-Smith H., Song Y., Tuan R.S. (2009). Interleukin-6 maintains bone marrow-derived mesenchymal stem cell stemness by an ERK1/2-dependent mechanism. J. Cell Biochem..

[88] Dorronsoro A., Lang V., Ferrin I., Fernández-Rueda J., Zabaleta L., Pérez-Ruiz E., Sepúlveda P., Trigueros C. (2020). Intracellular role of IL-6 in mesenchymal stromal cell immunosuppression and proliferation. Sci. Rep..

[89] Nakanishi M., Rosenberg D.W. (2013). Multifaceted roles of PGE2 in inflammation and cancer. Semin. Immunopathol..

[90] Lu X., Han J., Xu X., Xu J., Liu L., Huang Y., Yang Y., Qiu H. (2017). PGE2 promotes the migration of mesenchymal stem cells through the activation of FAK and ERK1/2 pathway. Stem Cells Int..

[91] Tsirvouli E., Noël V., Flobak Å., Calzone L., Kuiper M. (2024). Dynamic Boolean modeling of molecular and cellular interactions in psoriasis predicts drug target candidates. iScience.

[92] Chizzolini C., Brembilla N.C. (2009). Prostaglandin E2: Igniting the fire. Immunol. Cell Biol..

[93] Boniface K., Bak-Jensen K.S., Li Y., Blumenschein W.M., McGeachy M.J., McClanahan T.K., McKenzie B.S., Kastelein R.A., Cua D.J., Malefyt R.d.W. (2009). Prostaglandin E2 regulates Th17 cell differentiation and function through cyclic AMP and EP2/EP4 receptor signaling. J. Exp. Med..

[94] Su J., Chen X., Huang Y., Li W., Li J., Cao K., Cao G., Zhang L., Li F., Roberts A.I. (2014). Phylogenetic distinction of iNOS and IDO function in mesenchymal stem cell-mediated immunosuppression in mammalian species. Cell Death Differ..

[95] Kulesza A., Paczek L., Burdzinska A. (2023). The role of COX-2 and PGE2 in the regulation of immunomodulation and other functions of mesenchymal stromal cells. Biomedicines.

[96] Seo Y., Ahn J.-S., Shin Y.Y., Oh S.-J., Song M.-H., Kang M.-J., Oh J.-M., Lee D., Kim Y.H., Lee B.-C. (2022). Mesenchymal stem cells target microglia via galectin-1 production to rescue aged mice from olfactory dysfunction. Biomed. Pharmacother..

[97] Cao Z., Xie Y., Yu L., Li Y., Wang Y. (2020). Hepatocyte growth factor (HGF) and stem cell factor (SCF) maintained the stemness of human bone marrow mesenchymal stem cells (hBMSCs) during long-term expansion by preserving mitochondrial function via the PI3K/AKT, ERK1/2, and STAT3 signaling pathways. Stem Cell Res. Ther..

[98] Romano B., Elangovan S., Erreni M., Sala E., Petti L., Kunderfranco P., Massimino L., Restelli S., Sinha S., Lucchetti D. (2019). TNF-stimulated gene-6 is a key regulator in switching stemness and biological properties of mesenchymal stem cells. Stem Cells.

[99] Payne N.L., Sun G., McDonald C., Moussa L., Emerson-Webber A., Loisel-Meyer S., Medin J.A., Siatskas C., Bernard C. (2013). Human adipose-derived mesenchymal stem cells engineered to secrete IL-10 inhibit APC function and limit CNS autoimmunity. Brain Behav. Immun..

[100] El-Zayadi A.A., Jones E.A., Churchman S.M., Baboolal T.G., Cuthbert R.J., El-Jawhari J.J., Badawy A.M., Alase A.A., El-Sherbiny Y.M., McGonagle D. (2017). Interleukin-22 drives the proliferation, migration and osteogenic differentiation of mesenchymal stem cells: A novel cytokine that could contribute to new bone formation in spondyloarthropathies. Rheumatology.

[101] Yoo K.H., Jang I.K., Lee M.W., Kim H.E., Yang M.S., Eom Y., Lee J.E., Kim Y.J., Yang S.K., Jung H.L. (2009). Comparison of immunomodulatory properties of mesenchymal stem cells derived from adult human tissues. Cell Immunol..

[102] Chen M., Peng J., Xie Q., Xiao N., Su X., Mei H., Lu Y., Zhou J., Dai Y., Wang S. (2019). Mesenchymal stem cells alleviate moderate-to-severe psoriasis by reducing the production of type I interferon (IFN-I) by plasmacytoid dendritic cells (pDCs). Stem Cells Int..

[103] Cuesta-Gomez N., Medina-Ruiz L., Graham G.J., Campbell J.D.M. (2023). IL-6 and TGF-β-secreting adoptively-transferred murine mesenchymal stromal cells accelerate healing of psoriasis-like skin inflammation and upregulate IL-17A and TGF-β. Int. J. Mol. Sci..

[104] Sivanathan K.N., Rojas-Canales D., Grey S.T., Gronthos S., Coates P.T. (2017). Transcriptome profiling of IL-17A preactivated mesenchymal stem cells: A comparative study to unmodified and IFN- γ modified mesenchymal stem cells. Stem Cells Int..

[105] Du-Rocher B., Binato R., de-Freitas-Junior J.C.M., Corrêa S., Mencalha A.L., Morgado-Díaz J.A., Abdelhay E. (2020). IL-17 triggers invasive and migratory properties in human MSCs, while IFNy favors their immunosuppressive capabilities: Implications for the “licensing” process. Stem Cell Rev. Rep..

[106] Lee Y.S., Sah S.K., Lee J.H., Seo K.-W., Kang K.-S., Kim T.-H. (2016). Human umbilical cord blood-derived mesenchymal stem cells ameliorate psoriasis-like skin inflammation in mice. Biochem. Biophys. Rep..

[107] Duan X., Liu X., Liu N., Huang Y., Jin Z., Zhang S., Ming Z., Chen H. (2020). Inhibition of keratinocyte necroptosis mediated by RIPK1/RIPK3/MLKL provides a protective effect against psoriatic inflammation. Cell Death Dis..

[108] Bai L., Fang H., Xia S., Zhang R., Li L., Ochando J., Xu J., Ding Y. (2018). STAT1 activation represses IL-22 gene expression and psoriasis pathogenesis. Biochem. Biophys. Res. Commun..

[109] Li H., Wang X., Zhu J., Yang B., Lou J. (2024). Identifying key inflammatory genes in psoriasis via weighted gene co-expression network analysis: Potential targets for therapy. Biomol. Biomed..

[110] Korbecki J., Maruszewska A., Bosiacki M., Chlubek D., Baranowska-Bosiacka I. (2022). The potential importance of CXCL1 in the physiological state and in noncancer diseases of the cardiovascular system, respiratory system and skin. Int. J. Mol. Sci..

[111] Wang L., Wu Y., Yao R., Li Y., Wei Y., Cao Y., Zhang Z., Wu M., Zhu H., Yao Y. (2023). The role of mesenchymal stem cell-derived extracellular vesicles in inflammation-associated programmed cell death. Nanotoday.

[112] Liu X., Ren S., Qu X., Ge C., Cheng K., Zhao R.C.H. (2015). Mesenchymal stem cells inhibit Th17 cells differentiation via IFN-γ-mediated SOCS3 activation. Immunol. Res..

[113] Ding Y., Gong P., Jiang J., Feng C., Li Y., Su X., Bai X., Xu C., Liu C., Yang J. (2022). Mesenchymal stem/stromal cells primed by inflammatory cytokines alleviate psoriasis-like inflammation via the TSG-6-neutrophil axis. Cell Death Dis..

[114] Coughlan C., Bruce K., Burgy O., Boyd T.D., Michel C.R., Garcia-Perez J.E., Adame V., Anton P., Bettcher B.M., Chial H.J. (2020). Exosome isolation by ultracentrifugation and precipitation: A comparison of techniques for downstream analyses. Curr. Protoc. Cell Biol..

[115] Al-Khawaga S., Abdelalim E.M. (2020). Potential application of mesenchymal stem cells and their exosomes in lung injury: An emerging therapeutic option for COVID-19 patients. Stem Cell Res. Ther..

[116] Théry C., Zitvogel L., Amigorena S. (2002). Exosomes: Composition, biogenesis and function. Nat. Rev. Immunol..

[117] Kalluri R., LeBleu V.S. (2020). The biology, function, and biomedical applications of exosomes. Science.

[118] Li M., Li S., Du C., Zhang Y., Li Y., Chu L., Han X., Galons H., Zhang Y., Sun H. (2020). Exosomes from different cells: Characteristics, modifications, and therapeutic applications. Eur. J. Med. Chem..

[119] Koken G.Y., Abamor E.S., Allahverdiyev A., Karaoz E. (2022). Wharton jelly derived mesenchymal stem cell’s exosomes demonstrate significant antileishmanial and wound healing effects in combination with aloe-emodin: An in vitro study. J. Pharm. Sci..

[120] Zhou Y., Seo J., Tu S., Nanmo A., Kageyama T., Fukuda J. (2024). Exosomes for hair growth and regeneration. J. Biosci. Bioeng..

[121] Liu Y., Wang H., Wang J. (2018). Exosomes as a novel pathway for regulating development and diseases of the skin. Biomed. Rep..

[122] Lotfy A., AboQuella N.M., Wang H. (2023). Mesenchymal stromal/stem cell (MSC)-derived exosomes in clinical trials. Stem Cell Res. Ther..

[123] Li S., Liu J., Liu S., Jiao W., Wang X. (2021). Mesenchymal stem cell-derived extracellular vesicles prevent the development of osteoarthritis via the circHIPK3/miR-124-3p/MYH9 axis. J. Nanobiotechnol..

[124] de Almeida Fuzeta M., Bernardes N., Oliveira F.D., Costa A.C., Fernandes-Platzgummer A., Paulo Farinha J., Rodrigues C.A.V., Jung S., Tseng R.-J., Milligan W. (2020). Scalable production of human mesenchymal stromal cell-derived extracellular vesicles under serum-/xeno-free conditions in a microcarrier-based bioreactor culture system. Front. Cell Dev. Biol..

[125] Zhang N., Song Y., Huang Z., Chen J., Tan H., Yang H., Fan M., Li Q., Wang Q., Gao J. (2020). Monocyte mimics improve mesenchymal stem cell-derived extracellular vesicle homing in a mouse MI/RI model. Biomaterials.

[126] Wei X., Liu C., Wang H., Wang L., Xiao F., Guo Z., Zhang H., Camussi G. (2016). Surface phosphatidylserine is responsible for the internalization on microvesicles derived from hypoxia-induced human bone marrow mesenchymal stem cells into human endothelial cells. PLoS ONE.

[127] González-Cubero E., González-Fernández M.L., Gutiérrez-Velasco L., Navarro-Ramírez E., Villar-Suárez V. (2021). Isolation and characterization of exosomes from adipose tissue-derived mesenchymal stem cells. J. Anat..

[128] Kolenc A., Maličev E. (2024). Current methods for analysing mesenchymal stem cell-derived extracellular vesicles. Int. J. Mol. Sci..

[129] Tang Y., Zhou Y., Li H.J. (2021). Advances in mesenchymal stem cell exosomes: A review. Stem Cell Res. Ther..

[130] Hua C., Chen S., Cheng H. (2022). Therapeutic potential of mesenchymal stem cells for refractory inflammatory and immune skin diseases. Hum. Vaccin. Immunother..

[131] Michalak-Stoma A., Bartosińska J., Kowal M., Juszkiewicz-Borowiec M., Gerkowicz A., Chodorowska G. (2013). Serum levels of selected Th17 and Th22 cytokines in psoriatic patients. Dis. Markers.

[132] Qin S., Wen J., Bai X.-C., Chen T.-Y., Zheng R.-C., Zhou G.-B. (2014). Endogenous n-3 polyunsaturated fatty acids protect against imiquimod-induced psoriasis-like inflammation via the IL-17/IL-23 axis. Mol. Med. Rep..

[133] Nograles K.E., Davidovici B., Krueger J.G. (2010). New insights in the immunologic basis of psoriasis. Semin. Cutan. Med. Surg..

[134] Zhao J., Di T., Wang Y., Liu X., Liang D., Zhang G., Li P. (2016). Multi-glycoside of Tripterygium wilfordii Hook. f. ameliorates imiquimod-induced skin lesions through a STAT3-dependent mechanism involving the inhibition of Th17-mediated inflammatory responses. Int. J. Mol. Med..

[135] Martin D.A., Towne J.E., Kricorian G., Klekotka P., Gudjonsson J.E., Krueger J.G., Russell C.B. (2013). The emerging role of IL-17 in the pathogenesis of psoriasis: Preclinical and clinical findings. J. Investig. Dermatol..

[136] Shi X., Jin L., Dang E., Chang T., Feng Z., Liu Y., Wang G. (2011). IL-17A upregulates keratin 17 expression in keratinocytes through STAT1- and STAT3-dependent mechanisms. J. Investig. Dermatol..

[137] Rizzo H.L., Kagami S., Phillips K.G., Kurtz S.E., Jacques S.L., Blauvelt A. (2011). IL-23-mediated psoriasis-like epidermal hyperplasia is dependent on IL-17A. J. Immunol..

[138] Arican O., Aral M., Sasmaz S., Ciragil P. (2005). Serum levels of TNF-alpha, IFN-gamma, IL-6, IL-8, IL-12, IL-17, and IL-18 in patients with active psoriasis and correlation with disease severity. Mediat. Inflamm..

[139] Di Cesare A., Di Meglio P., Nestle F.O. (2009). The IL-23/Th17 axis in the immunopathogenesis of psoriasis. J. Investig. Dermatol..

[140] Zenewicz L.A., Flavel R.A. (2011). Recent advances in IL-22 biology. Int. Immunol..

[141] Van Belle A.B., de Heusch M., Lemaire M.M., Hendrickx E., Warnier G., Dunussi-Joannopoulos K., Fouser L.A., Renauld J.-C., Dumoutier L. (2012). IL-22 is required for imiquimod-induced psoriasiform skin inflammation in mice. J. Immunol..

[142] El Malki K., Karbach S.H., Huppert J., Zayoud M., Reissig S., Schüler R., Nikolaev A., Karram K., Münzel T., Kuhlmann C.R. (2013). An alternative pathway of imiquimod-induced psoriasis-like skin inflammation in the absence of interleukin-17 receptor a signaling. J. Investig. Dermatol..

[143] Mabuchi T., Takekoshi T., Hwang S.T. (2011). Epidermal CCR6+ γδ T cells are major producers of IL-22 and IL-17 in a murine model of psoriasiform dermatitis. J. Immunol..

[144] Dairov A., Sekenova A., Alimbek S., Nurkina A., Shakhatbayev M., Kumasheva V., Kuanysh S., Adish Z., Issabekova A., Ogay V. (2024). Psoriasis: The versatility of mesenchymal stem cell and exosome therapies. Biomolecules.

[145] Ye Z., Liang Y., Lin B., Li Y., Chai X., Lian J., Zhang X., Che Z., Zeng J., Pillai G. (2022). Gingiva-derived mesenchymal stem cells attenuate imiquimod- (IMQ-) induced murine psoriasis-like skin inflammation. Stem Cells Int..

[146] Zhang Y., Kim T.-J., Wroblewska J.A., Tesic V., Upadhyay V., Weichselbaum R.R., Tumanov A.V., Tang H., Guo X., Tang H. (2018). Type 3 innate lymphoid cell-derived lymphotoxin prevents microbiota-dependent inflammation. Cell Mol. Immunol..

[147] Balato N., Napolitano M., Ayala F., Patruno C., Megna M., Tarantino G. (2015). Nonalcoholic fatty liver disease, spleen and psoriasis: New aspects of low-grade chronic inflammation. World J. Gastroenterol..

[148] Müller L., Tunger A., Wobus M., von Bonin M., Towers R., Bornhäuser M., Dazzi F., Wehner R., Schmitz M. (2021). Immunomodulatory properties of mesenchymal stromal cells: An update. Front. Cell Dev. Biol..

[149] Cai Y., Fleming C., Yan J. (2012). New insights of T cells in the pathogenesis of psoriasis. Cell Mol. Immunol..

[150] Zhang P., Su Y., Li S., Chen H., Wu R., Wu H. (2023). The roles of T cells in psoriasis. Front. Immunol..

[151] Nussbaum L., Chen Y.L., Ogg G.S. (2021). Role of regulatory T cells in psoriasis pathogenesis and treatment. Br. J. Dermatol..

