# Supplementary figures and images for: Study of the Therapeutic Effect of Cytokine-Preconditioned Mesenchymal Stem Cells and Their Exosomes in a Mouse Model of Psoriasis
Source: Biology (Basel). 2025 Aug 11;14(8):1033. doi: 10.3390/biology14081033 (PMC12383953; doi:10.3390/biology14081033)

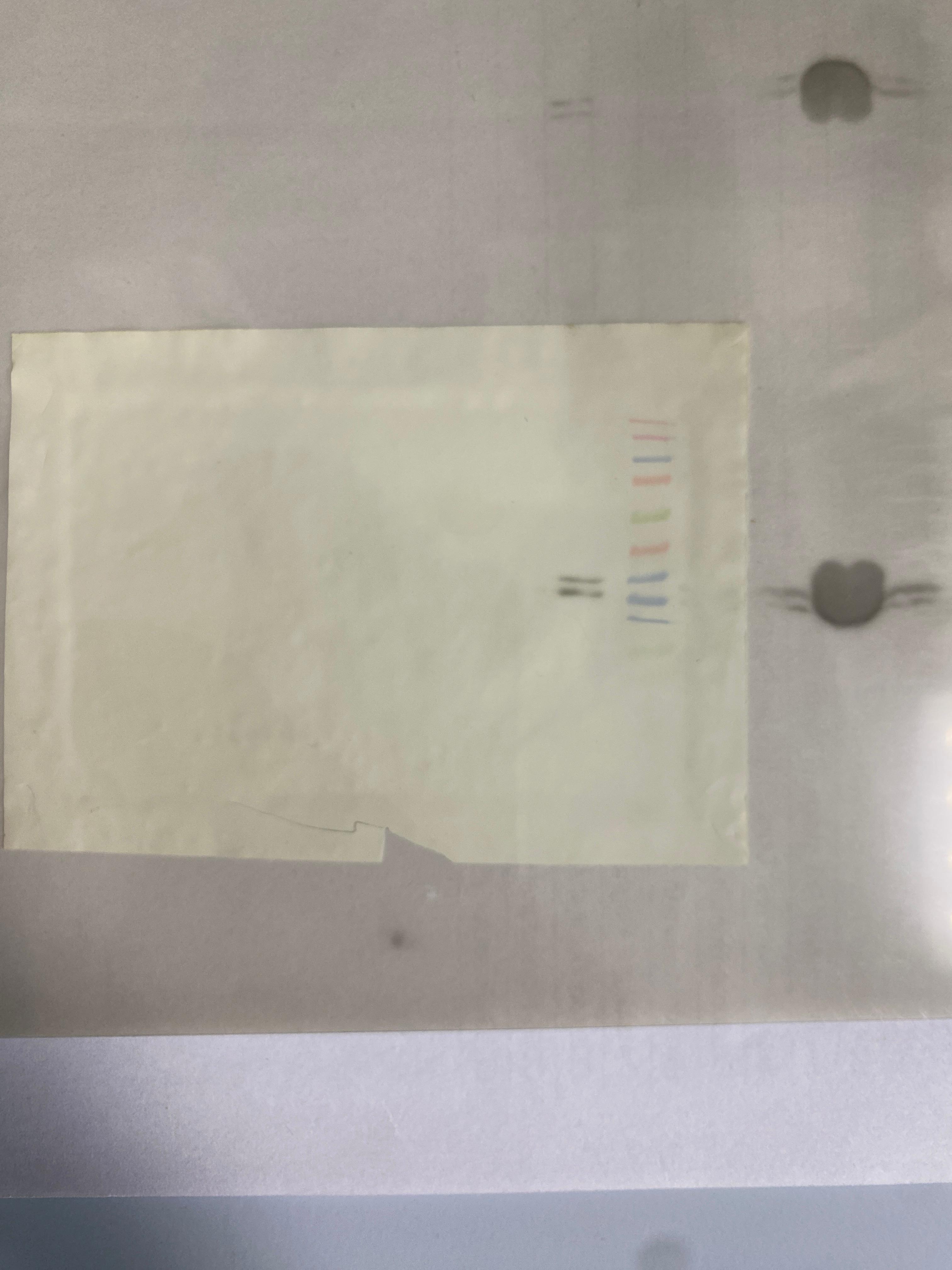

Supplement: Supplementary file 1 [file biology-14-01033-s001.zip › biology-3768616-supplementary.jpeg]
